# The Symptoms and Impacts Experienced by Healthcare Professionals as Second Victims After a Safety Incident: A Scoping Review

**DOI:** 10.1111/jan.70196

**Published:** 2025-09-30

**Authors:** Laura Jukarainen, Sanu Mahat, Saija Koskiniemi, Tiina Syyrilä, Albert W. Wu, Virpi Jylhä, Marja Härkänen

**Affiliations:** ^1^ Department of Nursing Science University of Eastern Finland (UEF) Kuopio Finland; ^2^ The Finnish Centre for Evidence‐Based Health Care: A JBI Centre of Excellence Helsinki Finland; ^3^ Johns Hopkins Bloomberg School of Public Health Johns Hopkins School of Medicine Baltimore Maryland USA; ^4^ Research Centre for Nursing Science and Social and Health Management, Kuopio University Hospital Wellbeing Services County of North Savo Kuopio Finland; ^5^ Department of Social and Health Management University of Eastern Finland Kuopio Finland

**Keywords:** healthcare professional, safety incident, scoping review, second victim

## Abstract

**Aim:**

This study aimed to describe the types of psychological and physical symptoms experienced by healthcare professionals who became second victims after a patient safety incident and the impact of the incident on their social and professional lives.

**Design:**

Scoping review.

**Methods:**

JBI methodology for scoping reviews and PRISMA‐ScR for reporting were followed.

**Data Sources:**

The search was conducted on June 13, 2024, using the CINAHL (EBSCO), Scopus, PubMed (Medline), Medic and PsycInfo (EBSCO) databases. A grey literature search was also conducted.

**Results:**

A total of 96 papers were included. Healthcare professionals experienced psychological symptoms such as anger, sadness and guilt after a safety incident. Physical symptoms were reported, including symptoms related to sleep and gastrointestinal symptoms. At the professional and social levels, the incident affected their work, relationships and well‐being. Positive impacts were also noted.

**Conclusions:**

This study provides a comprehensive overview of healthcare professionals' experiences after safety incidents. In addition, this study also captured the positive impacts of safety incidents, such as learning from mistakes.

**Implications for the Profession and/or Patient Care:**

By recognising the symptoms and impacts associated with the second victim syndrome, appropriate support can be provided for healthcare professionals.

**Impact:**

The findings of this study can be used to identify the relevant harm to professionals after a safety incident, which could help to improve the well‐being of these workers.

**Patient or Public Contribution:**

No patient or public contribution.

**Protocol Registration:**

Open Science Framework, https://archive.org/details/osf‐registrations‐5cdmu‐v1

**Summary**

- What does this paper contribute to the wider global clinical community?
  ○ When healthcare professionals' experienced symptoms, as well as professional and social impacts, can be identified, appropriate assistance can be targeted to them. As a result, the quality and safety of healthcare can be improved.

## 1 Introduction

The World Health Organization (WHO) defines a safety incident as an event that can cause harm to a patient. A near‐miss safety incident refers to a situation in which there is a risk of harm, but it does not affect the patient. A no harm incident is one where the incident reached the patient, but there was no harm to the patient. A harmful incident indicates that the patient experienced harm from the event. An adverse event is defined as an event that caused preventable harm to the patient (WHO 2020). This scoping review includes all types of safety incidents, including near‐miss and harmful incidents.

The term ‘second victim’ (SV) was introduced to describe the harm experienced by a healthcare professional (HCP) involved in an incident. The first victim refers to the patient who has been involved. The SV term was introduced by Wu (2000). The SV term has also received criticism. Referring to an HCP involved in a safety incident as an ‘SV’ may divert attention away from the patient and their loved ones, who are the first victims of the incident. Based on the criticism, referring to an HCP as a victim after a safety incident may reduce the emphasis on their professional responsibility for what happened. It is argued that the use of the term ‘SV’ obscures the fact that HCPs and organisations can indeed cause harm to patients. The term is perceived as a potential threat to the advancement of patient safety and the process of learning from safety incidents (Clarkson et al. 2019). The term third victim has also been identified, referring to the organisation where the incident occurred (MacLeod 2014).

SVs are more likely to experience absenteeism from work and have high turnover intentions (Mahat et al. 2024). Second victim syndrome (SVS) describes the trauma experienced by an HCP after being involved in a safety incident. SVS affects various aspects of HCPs' lives (Ozeke et al. 2019). A German study found that up to 60% of nurses have experienced SVS, and up to 24% reported that recovery took more than a year (Strametz et al. 2021). Identifying the symptoms and impacts of safety incidents on HCPs is essential to help mitigate potential harm (Shuangjiang et al. 2022). The findings of this review can be utilised to identify harmful experiences of SVs, making it easier to direct appropriate support to them. This may help reduce professionals' absences from work and other potential consequences of safety incidents.

Safety incidents affect not only the patient but also the personal and professional lives of HCPs in various ways, leading to psychological, emotional, behavioural, social and physical consequences (Buhlmann et al. 2021; Kappes et al. 2021). SVS is often associated with distress, guilt and other psychological symptoms resulting from safety incidents. Physical symptoms such as sleep disturbance, fatigue and elevated heart rate may also occur (Mitzman et al. 2019). Safety incidents can impact the professional life of SVs. The loss of professional confidence is common among SVs. HCPs may encounter social challenges following safety incidents (Awuah et al. 2024).

After a safety event, professionals may also experience increased empathy and understanding towards their colleagues. They also want to share what happened, especially with colleagues who are in a similar situation (Brunelli et al. 2024). Beyond advancing patient safety, learning from errors and strengthening evidence‐based practice are also essential for supporting HCPs after a safety incident (Peddle et al. 2024). So far, the positive impacts experienced by HCPs following a safety incident have been studied to a limited extent. This review also seeks to identify the positive impacts.

Previous studies have focused on identifying the most common SVS‐related symptoms (Sachs and Wheaton 2024), exploring the SVS among nursing students and nurses (Sahay and McKenna 2023) as well as in obstetrics (Nydoo et al. 2020), describing nurses' psychological responses and coping mechanisms as SVs (Chan et al. 2017), examining organisational and personal factors that help HCPs navigate the SVS (Kappes et al. 2021) and analysing HCPs psychological and psychosomatic symptoms after an adverse event (Busch et al. 2020). Previous scoping reviews have focused on validating the Second Victim Experience and Support Tool (Dato Md Yusof et al. 2023), peer support (Carbone et al. 2022), the SVS and organisational support (Petryszyn et al. 2023). One systematic review examined SV terminologies, definitions, prevalence and coping strategies (Seys et al. 2013).

Published systematic review protocols have focused on physicians (Laetitia Gimenez et al. 2020) and the prevalence of psychosomatic symptoms, psychological responses and coping strategies among HCPs regarding patient safety incidents (Busch et al. 2020). Additionally, studies and published protocols have focused on nurses' and midwives' SV experiences and responses following an error (Carr et al. 2018), feelings of being a SV among Spanish obstetricians and midwives (Santana‐Domínguez et al. 2022), HCPs SV experiences after an adverse event (Botero Carvajal Alejandro et al. 2021) and SVS among intensive care unit healthcare workers (Naya et al. 2023). However, none of the published scoping reviews have specifically addressed both the symptoms associated with safety incidents and the impacts they can have on HCPs.

The WHO published the Global Safety Action Plan in 2021, stating that HCPs who are both mentally and physically healthy make fewer errors in patient care (WHO 2021). Exploring the symptoms and impacts is crucial in developing strategies to help HCPs cope with SVS. By supporting professionals and promoting their mental and physical health after a safety incident, patient safety can be improved. Previous reviews have not comprehensively compiled both the psychological and physical symptoms, as well as the social and professional impacts on healthcare professionals. This scoping review differs from previous reviews in that it considers all types of safety incidents and includes all HCPs. Furthermore, it examines the impact of safety incidents on the social and professional lives of HCPs, in addition to physical and psychological symptoms.

### 1.1 Aims

This study aimed to describe the types of psychological and physical symptoms experienced by HCPs who became SVs after a patient safety incident and the impact of the incident on their social and professional lives.

### 1.2 Review Questions

1. What psychological symptoms do HCPs experience after a safety incident?
2. What physical symptoms do HCPs experience after a safety incident?
3. How do safety incidents impact the social and professional lives of HCPs?

## 2 Methods

### 2.1 Design

This is an evidence synthesis study by using a scoping review, which is a valid method for clarifying key concepts and understanding and mapping existing evidence from the literature that has already been studied (Munn et al. 2018). This study aimed to explore the types of evidence on SVS symptoms and their impacts, so a scoping review is a justified methodological choice (Pollock et al. 2023).

This study was conducted according to the JBI methodology for scoping reviews (Aromataris et al. 2024). The review followed the guidelines of the Preferred Reporting Items for Systematic Reviews and Meta‐analyses Extension for Scoping Reviews (Prisma‐ScR) checklist (Tricco et al. 2018). The title, aim, review questions and inclusion criteria were registered within Open Science Framework, published on June 5, 2024 (registration number 5CDMU).

### 2.2 Search Strategy

The search strategy aimed to locate both published and unpublished studies as well as grey literature. A three‐step strategy was implemented in this review. The MeSH (Medical Subject Headings) vocabulary thesaurus was used to generate appropriate search terms for PubMed (Medline). Additionally, an information specialist contributed to developing an appropriate search phrase. The initial limited search of the PubMed (Medline), Medic, and CINAHL (EBSCO) scientific databases was conducted to identify relevant articles on the topic on March 5, 2024 (Table 1). The search phrase was (“Health Personnel” OR “nurse*” OR “nursing staff” OR “medical staff” OR “Health Personnel” OR “health professional*”) AND (“second victim*”). The same search phrase was used across all the scientific databases utilised. In the search, the term ‘SV’ was used to restrict the search specifically to healthcare professionals affected by patient safety incidents. The final data search was performed using the same search phrase in CINAHL (EBSCO), Scopus, PubMed (Medline), Medic and PsycInfo on June 13, 2024. Inclusion criteria were Finnish or English language due to resource availability and language proficiency. The time period was limited to studies published between 2000 and 2024. The search time frame was broad to ensure comprehensive coverage, as the SV concept became widely recognised in the 21st century (Wu 2000).

**TABLE 1:** Scientific searches to databases (Year limits: 2000–2024).

Search	Query	Results
PubMed (Medline)		
#5	#1 AND #2 Filters: English, Finnish	220
#4	#1 AND #2 Filters: Finnish	0
#3	#1 AND #2	235
#2	(“second victim”)	376
#1	(“Health Personnel” OR “nurse*” OR “nursing staff” OR “medical staff” OR “health professional”)	762,471
CINAHL (EBSCO)		
#4	#1 AND #2 Filters:English	154
#3	#1 AND #2	167
#2	(“second victim”)	236
#1	(“Health Personnel” OR “nurse*” OR “nursing staff” OR “medical staff” OR “health professional”)	684,850
Scopus		
#3	#1 AND #2	283
#2	(“second victim”)	565
#1	(“Health Personnel” OR “nurse*” OR “nursing staff” OR “medical staff” OR “health professional”)	875,140
PsycInfo		
#3	#1 AND #2 Filters: English	51
#2	(“second victim”)	89
#1	(“Health Personnel” OR “nurse*” OR “nursing staff” OR “medical staff” OR “health professional”)	232,843

To find grey literature, a search was conducted relevant on websites between 14 and 16 June 2024 and suitable articles were manually searched within reference lists. The selection of websites was based on the subject‐matter expertise of the authors. The following websites were used: The European Researchers' Network Working on Second Victims (ERNST), The Agency for Healthcare Research and Quality (AHRQ), the International Society for Quality in Health Care (ISQua), and The National Health Services in UK (NHS). These websites were selected because they are well‐known official sources in the healthcare field and publish papers and information broadly related to healthcare topics. Websites were searched using search boxes. The search term used was ‘second victim’. The search terms were intentionally kept simple to maximise relevant results. The author reviewed all results found through the website search. These results were manually compared with those from the scientific database search to determine whether the findings from the websites were already identified through the scientific search. The scientific database searches and corresponding search phrases used are presented in Table 1.

### 2.3 Inclusion and Exclusion Criteria

This review utilises the PCC mnemonic. The acronym PCC stands for population, concept and context (Peters et al. 2021).

#### 2.3.1 Population

The population in this scoping review was HCPs who had been involved in any type of safety incident, including near misses and incidents causing harm. For the purposes of this scoping review, HCPs were defined as medical doctors, registered nurses, licenced nurses, practical nurses, midwives, pharmacists, dentists and paramedical practitioners (WHO 2013) involved in safety incidents. Papers that focused on professionals who had not experienced a safety incident were excluded.

#### 2.3.2 Concept

The concept encompasses SVS‐related psychological and physical symptoms after a patient safety incident, as well as their impact on the social and professional lives of HCPs. Studies focusing only on SV support methods after the incident were excluded. However, all studies examining SV symptoms and safety incidents impacts on social or professional life were included.

#### 2.3.3 Context

This review focuses on the healthcare sector. For the purposes of this scoping review, healthcare is defined as organised medical care for individuals and communities (Faulkner and Nicholson 2020). For the purposes of this scoping review, the healthcare context was broad and included community health as well as primary, secondary and tertiary care units (NHS 2022). Healthcare systems from low‐, middle‐, and high‐income countries were included. Digital health services were also considered in this study.

#### 2.3.4 Types of Sources

This scoping review considered both experimental and quasi‐experimental study designs such as randomised controlled trials, nonrandomised controlled trials, before and after studies and interrupted time‐series studies. In addition, analytical observational studies, such as prospective and retrospective cohort, case–control and analytical cross‐sectional studies, were considered for inclusion. This review also considered descriptive observational study designs, such as case series, individual case reports and cross‐sectional studies. Qualitative studies such as those limited to phenomenology, grounded theory, ethnography, qualitative description and action research were also considered. In addition, all reviews that met the inclusion criteria were considered, depending on the research question. In scoping reviews, it is justified to include a wide variety of literature, such as grey literature (Peters et al. 2021). A range of sources such as discussion papers, texts and opinion papers, books, websites related to the topic, editorials, conference papers, policies, commentaries and other relevant papers were considered in this scoping review.

### 2.4 Study Selection

**FIGURE 1:**
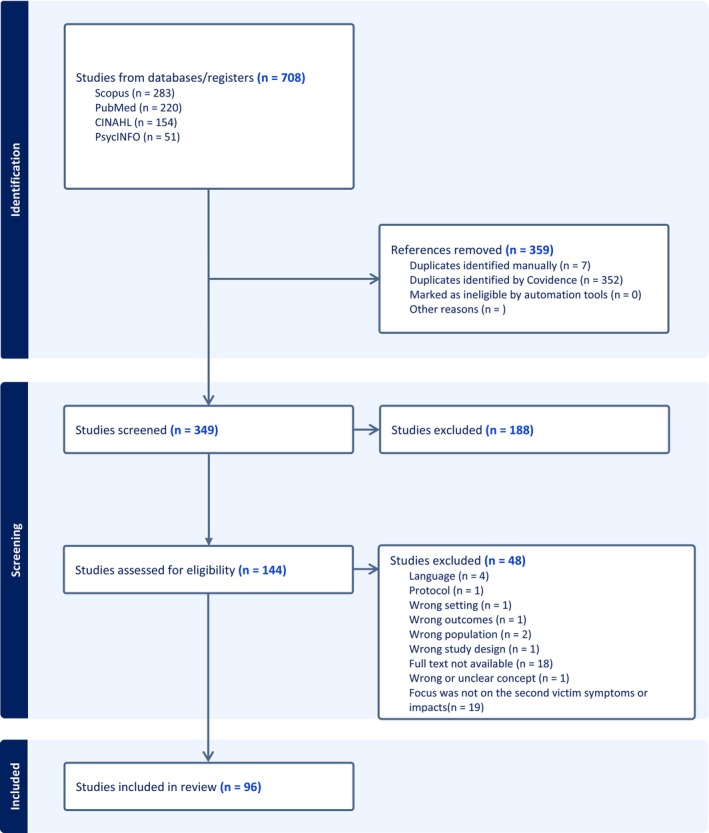
Flow chart. Healthcare professionals' psychological and physical symptoms after a safety incident and understanding its social and professional impacts: A scoping review.

Following an electronic search of the databases, all identified papers (*n* = 708) were collated and uploaded to the Covidence database (Veritas Health Innovation, Melbourne, Australia), and duplicates were removed. The grey literature search results were excluded due to duplicates of scientific papers. After the search, the titles and abstracts of 349 papers were screened by the first and the second author for assessment against the inclusion criteria for the review. After examining the titles and abstracts, the first and the fourth author read the full texts (*n* = 144) and screened them against the eligibility criteria. Disagreements between the authors were resolved by discussion or by consulting the last author. The screening process of the papers was continued until full consensus was reached. The results of the data search and article selection are shown in the PRISMA flowchart (Figure 1).

### 2.5 Data Extraction

The first author and the fourth author extracted data from the papers using a data extraction tool developed by the reviewers. Three authors, the first author, the fourth author and the last author, piloted the data extraction template. During the piloting of the form, the three authors reviewed its content item by item. Each author piloted three separate papers. The piloting process involved evaluating the items included in the form, and the authors were also able to add additional comments. Based on the feedback received during the piloting, minor changes were made to the wording used in the form. The data extraction was done by the first and the second author. The extracted data included specific details including the title, publication year, authors, journal, country where the paper was conducted, aims, type of evidence, design, methods, sample, date range when the research or paper was conducted, participants, context and key findings relevant to the review questions. Disagreements were resolved by the last author. The data extraction form is outlined in Table 2. The quality of the selected papers was not analysed in accordance with the methodology guidelines of the JBI for scoping reviews (Peters et al. 2021).

**TABLE 2:** Data extraction instrument.

Data details	
Title	
Authors	
Publication year	
Journal or website	
Country in which the study conducted	Australia Brazil China Denmark Germany Iran Israel Italy Korea Netherlands Spain Sweden United Kingdom United States Other _____
Aims/objectives	
2 Information of the study	
Type of evidence	Research articles Synthesis of the evidence Grey literature Other _____
Design	Quantitative Qualitative Quantitative and qualitative Longitudinal Cross‐sectional Retrospective Prospective Multimethod Other _____
Methods	Experimental Observational Case–control Case report Case series RCT Descriptive Cohort Systematic review Scoping review Literature review Mixed methods Ethnographic Prevalence Survey Interview Other ______
Sample and sample size	
Date range (when the study or paper was conducted)	
Participants	Registered nurses Practical nurses Licenced nurses Nurses Dentists Pharmacists Physicians Medical doctors Midwives Paramedical practitioner Other _____
Context (healthcare)	Primary health care Secondary health care Tertiary health care Community health care Electronic health care Other ____
The results compared to the research questions	
What kind of psychological symptoms do healthcare professionals experience after a safety incident?	
What physical symptoms do healthcare professionals have after a safety incident?	
How does a safety incident impact the social and professional life of a healthcare professional?	

### 2.6 Data Synthesis

Data were categorised using deductive and inductive methods. Psychological symptoms were categorised deductively using the results of the article by Mahat et al. (2022) as a framework. Psychological symptoms categorised deductively were feelings of fear and anxiety (Mahat et al. 2022), anger (Mahat et al. 2022), feelings of sadness (Mahat et al. 2022), feelings of guilt (Mahat et al. 2022), feeling disturbed (Mahat et al. 2022), feelings of shame (Mahat et al. 2022), depression (Mahat et al. 2022), self‐esteem (Mahat et al. 2022). Data that could not be categorised using the deductive method were inductively categorised. Inductively categorised psychological symptoms were positive psychological symptoms and reactions, and other psychological reactions and responses. The symptoms and impacts of the papers were organised into main categories and subcategories. The results were reported narratively. Word clouds were also created based on the results. The word clouds illustrate the most common physical symptoms, psychological symptoms and professional and social impacts experienced by professionals. As the review included multiple papers (*n* = 96), the citations in the text were limited to a maximum of three to enhance readability. All citations supporting the findings can be found in Table 9.

## 3 Results

### 3.1 Characteristics of Included Studies

A total of 96 papers were included in the scoping review. The characteristics of the included studies are presented in Table 3. All the included papers were published between 2009 and 2024. These papers were conducted across 25 countries. The largest number of papers in this scoping review was conducted in the United States (*n* = 30 papers). All countries are listed in Table 4. Most of the papers were original research (*n* = 63), while the rest were a synthesis of evidence (*n* = 21) and grey literature (*n* = 12). Grey literature was found through scientific search. Study designs included were quantitative (*n* = 43), qualitative (*n* = 36), mixed‐methods (*n* = 5), cross‐sectional (*n* = 40), descriptive (*n* = 18), retrospective (*n* = 2) and exploratory (*n* = 2). In addition, there was one grounded theory and one cross‐country study. The methods used included surveys (*n* = 45), interviews (*n* = 20), systematic reviews (*n* = 5), literature reviews (*n* = 10), observations (*n* = 2), scoping reviews (*n* = 2) and meta‐analyses (*n* = 2). Among the included studies, one study used text mining as a method. Grey literature papers were discussion papers (*n* = 7), editorials (*n* = 2), scientific letters, journal course and commentary articles.

**TABLE 3:** Characteristics of the included studies and papers.

Authors, year of publication and country	Paper type	Aims/objectives	Design and methods	Population	Concept	Context
1. Abd Elwahab and Doherty (2014). Ireland.	Discussion paper	To discuss the impact of medical errors on doctors, including the consequent stress and emotional effects, burnout and deteriorating professional performance.	N/A	N/A	The impact of medication errors on doctors.	N/A
2. Ajri‐Khameslou et al. (2017). Iran.	Original research	The aim was to interpret the causes that place nurses in danger of errors in emergency departments and also the consequences resulting from confronting the errors in the job environment.	Qualitative and in‐depth semi‐structured interviews	18 emergency nurses	The factors contributing to errors in emergency departments and their impact on nurses.	Educational hospitals
3. Alevi et al. (2024). Spain.	Original research	To describe the prevalence of newly graduated nurses as second victims of adverse events and to know the conditions of support received in health institutions.	Quantitative cross‐sectional survey	138 registered nurses	Newly graduated nurses second victim experiences after adverse events.	N/A
4. Amit Aharon et al. (2021). Israel.	Original research	To understand the effects of patients' suicidal attempts and events on nurses' second victim symptoms and to explore the association between these experiences and nurse absenteeism and turnover.	Mixed methods: a cross‐sectional quantitative survey and in‐depth interviews	150 nurses	Nurses' second victim symptoms after a patients' suicidal attempt.	Tertiary health care
5. Baas et al. (2018). Netherlands.	Original research	To study work‐related traumatic events and posttraumatic stress disorder among obstetricians gynaecologists and the (desired) type of support.	Quantitative cross‐sectional survey	683 gynaecologists	Gynaecologists and obstetricians' experiences of adverse events and these events impact to them.	N/A
6. Bakshi et al. (2022). United States.	Original research	This project evaluates the need for second victim resources in trauma care providers at a tertiary public level 1 trauma hospital	Quantitative cross‐sectional survey	35 nurses, doctors and advanced care professionals	Second victim syndrome among trauma care providers	Tertiary care
7. Bañeras et al. (2022). Spain.	Scientific letter	The aim was to investigate the experience of these healthcare providers as SVs and the quality of support resources in cardiology.	Quantitative cross‐sectional survey	198 nurses, physicians and residents.	Cardiology professionals second victim experiences after adverse events.	N/A
8. Buhlmann et al. (2022). Australia.	Original research	To gain a deeper understanding of nurses and midwives' experiences following involvement in a critical incident in a noncritical care area and to explore how they have ‘moved‐on’ from the event.	Qualitative interpretive design using semi‐structured interviews.	10 nurses and midwives.	Second victim experiences after a critical incident.	Noncritical care setting
9. Buhlmann et al. (2021). Australia.	Evidence synthesis	To synthesise the existing literature, which focuses on the impact of critical incidents on nurses and midwives, and to explore their experiences related to the support they received in the current healthcare environment to move on from the event.	Systematic review and qualitative synthesis.	11 qualitative primary research were included in systematic review.	Second victim experiences after a critical incident.	Tertiary care
10. Burlison et al. (2021). United States.	Original research	To assess the relationships between self‐reported second victim‐related distress to turnover intention and absenteeism.	Quantitative cross‐sectional survey	155 nurses	Second victim phenomenon, distress and job‐related outcomes.	Tertiary care
11. Busch et al. (2020). Italy.	Evidence synthesis	To provide a comprehensive synthesis and critical analysis of second victims' emotional distress.	Systematic review and meta‐analysis	18 studies were included in systematic review	Second victims psychological and psychosomatic symptoms after an adverse event	N/A
12. Cabilan and Kynoch (2017). Australia.	Evidence synthesis	To synthesise the best available evidence on nurses' experiences as second victims, and explore their experiences of the support they receive and the support they need.	Qualitative systematic review.	Nine qualitative studies were included in this systematic review.	Nurses second victim experiences after a adverse nursing errors.	Any healthcare settings worldwide.
13. Chan et al. (2018). Singapore.	Original research	The aim of the study was to explore the psychological responses, coping strategies and support needs of Singapore nurses as second victims of adverse events.	Qualitative descriptive study using individual interviews.	8 nurses.	Nurses psychological responses after an adverse event.	Tertiary care
14. Chan et al. (2017). Singapore.	Evidence synthesis	To provide an overview of healthcare professionals' psychological responses, coping strategies and supporting needs in the aftermath of an adverse event, thus informing health policy implications and future research in this aspect.	Qualitative, literature review.	30 studies were included in this review.	Healthcare professionals psychological responses after an adverse event.	N/A
15. Cho et al. (2022). Korea.	Original research	To evaluate colonoscopists' second victim experience and the perception discordance between colonoscopists and patients for the colonoscopic perforation.	Quantitative cross‐sectional survey	160 colonoscopists	Second victim experience after a colonoscopic perforation.	N/A
16. Choi et al. (2020). Korea.	Original research	The aim of this study was to investigate the scope and severity of the second victim problem among nurses by examining the experiences and effects of patient safety incidents (PSIs) on them.	Quantitative cross‐sectional survey	492 nurses	Nurses second victim experiences after a patient safety incident.	Medical institutions
17. Chong et al. (2024). Singapore.	Evidence synthesis	This scoping review aimed to consolidate existing studies pertaining to a surgeon's experience with SVS, by broadly examining the prevalence and impact, identifying the types of responses, and evaluating factors that could influence these responses.	Qualitative, scoping review	13 articles were included in this scoping review.	Second victim symptoms among surgeons.	Tertiary
18. Cohen et al. (2023). Israel.	Original research	Using the second victims' natural history of recovery model to examine the impact of the SVP on Israeli nurses, with a specific focus on the organisational support they felt they required compared with the support they felt that they had received from their organisations.	Descriptive qualitative approach, in‐depth interviews	15 nurses	Second victim experiences after an adverse event.	N/A
19. Coughlan et al. (2017). Ireland.	Evidence synthesis	This review aimed to study not only the phenomenon of second victim in general medical care but to also concentrate on maternity care where the expectation of perfection may be argued to be greater.	Qualitative, literature review	N/A	Second victim experiences in general medical and maternity care.	N/A
20. Delacroix (2017). United States.	Original research	To explore the experience of committing medical error from the perspective of nurse practitioners (NPs). Overall, the purpose of the study is to discern NPs' behaviours, perceptions and coping mechanisms in response to having made a medical error.	Qualitative research based on semi‐structured face to face interviews	10 nurse practitioners	Nurse practitioners experiences after a medical error.	N/A
21. Draus et al. (2022). United States.	Original research	To determine the prevalence of nurses who identified as SVs and their awareness and use of supportive resources.	Quantitative, cross‐sectional, descriptive survey	160 nurses	Nurses second victim perceptions.	Hospital setting
22. Marran (2019). United Kingdom.	Discussion paper	The aim of this article is to explore the concept of healthcare professionals as ‘second victims’.	N/A	N/A	The potential effects of being a second victim.	N/A
23. Ferrús et al. (2021). Spain.	Original research	Identify what occurs among healthcare providers (HCPs) after an adverse event (AE) and what colleagues could do to help them.	Qualitative, focus group and metaplan.	15 HCPs and 12 health (=27) professionals.	Healthcare professionals' experiences after an adverse event.	Primary healthcare and hospitals.
24. Finney et al. (2021). United States.	Original research	Specifically, this study aims to: (a) determine the prevalence and types of SVEs in nurses; (b) explore the types of support services used or most desired by nurses; and (c) identify risk factors for SVEs among nurses.	Quantitative cross‐sectional survey	115 nurses	Types of second victim experiences in nurses.	Department of obstetricts and gynaecology
25. Ganahl et al. (2022). Austria.	Original research	In order to obtain a deeper understanding of the natural course of second victim traumatization and the demand for support in high‐risk populations, such as intensive care nurses.	Qualitative, semi‐structured interviews	20 intensive care nurses	Second victim traumatization	Tertiary
26. Goncharuk et al. (2024). Croatia.	Original research	The aim of this study was to identify the extent to which various groups of healthcare professionals involved in adverse events developed symptoms of distress and whether they were asking for psychological help.	Quantitative cross‐sectional survey	93 nurses and doctors	Healthcare professionals as second victims after adverse events and symptoms they developed.	Tertiary
27. Hall and Scott (2012). United States.	Evidence synthesis	N/A	N/A	N/A	The second victim phenomenon after an adverse event.	N/A
28. Han et al. (2017). United States.	Original research	Article aimed to assess the surgeons' personal account of iAE incidence, emotional response to iAEs, most frequently used social support systems and perspective regarding the barriers to iAE reporting.	Quantitative cross‐sectional survey	126 surgeons	Surgeons' emotional reactions after an adverse event.	Tertiary
29. Harrison et al. (2015). United States, United Kingdom.	Original research	The objectives were: (a) the professional or personal disruption experienced after making an error, (b) the emotional response and coping strategies used, (c) the relationship between emotions and coping strategy selection, (d) influential factors in clinicians' responses and (e) perceptions of organisational support.	Quantitative, cross‐sectional, cross‐country survey	120 physicians and 145 nurses	Emotional reactions after an error	Tertiary
30. Huang et al. (2022). China.	Original research	To investigate the experience and support of nurses as second victims in adverse events and explore factors.	Quantitative cross‐sectional survey	2897 nurses	Nurses second victim experiences after an adverse event.	Tertiary
31. Jones and Treiber (2012). United States.	Discussion paper	In this paper, we analyse the concept of ‘second victim’ within the context of medication administration errors. We also examine factors that contribute to nurses becoming second victims after making an error.	N/A	N/A	Nurses as second victims after a medication administration erros.	N/A
32. Jones and Treiber (2018). United States.	Original research	This article presents a study designed to investigate perceptions of recent BSN graduates about preparation for medication administration, medication error and their personal experience with error making and second victimhood.	Descriptive cross‐sectional mixed‐methods study using survey	168 nurses (former students)	BSN graduates' perceptions about making an error and second victim phenomenon	N/A
33. Kable, Kelly, and Adams (2018). Australia.	Original research	The aim of the present study was to understand the effects of adverse events in health care on nurses in acute healthcare settings in an Australian context.	Qualitative descriptive study using interviews	10 nurses	Nurses' experiences after an adverse event	Acute care in regional area
34. Kappes et al. (2023). Chile.	Original research	The objective of this research is to determine the prevalence of second victim hood, focusing on psychological distress, among Chilean adult intensive care nurses and its relationship with the support provided by their organisations.	Descriptive quantitative cross‐sectional correlation study using survey	326 nurses	Prevalence of second victim hood and psychological distress	Tertiary
35. Kaur et al. (2019). United States.	Original research	Our goal was to gain understanding and raise awareness about the culture of blame and guilt regarding medical errors that exists among healthcare professionals working in critical care.	Quantitative descriptive survey	901 participants (physicians, resident physicians, nurse practitioners, physician assistants)	Errors and their impact to healthcare professionals	Tertiary
36. Krommer et al. (2023). Austria.	Original research	Therefore, this study aimed to (1) conduct a descriptive study on SVP in one location (KHI); (2) compare the results of the study to similar previous studies; and (3) help develop targeted organisational strategies on how to manage the SVP in the future.	Descriptive quantitative cross‐sectional monocentric study	966 participants (nurses, doctors, other medical technicians and occupations)	Healthcare professionals second victim phenomenon	Tertiary
37. Kruse et al. (2024). United States.	Original research	We aimed to determine the prevalence of the second victim experience among CRNAs and to determine which types of support are seen as most beneficial to promoting healing and adaptation.	Quantitative descriptive cross‐sectional survey	172 CRNAs	The second victim experience among CRNAs	Tertiary
38. Lee, Pyo, Jang, Choi, and Ock (2019). Korea.	Original research	The aim was to examine the experiences and response methods of second victims to PSIs.	Qualitative study using in‐depth interviews	16 participants (nurses, physicians, pharmacists)	Second victims' experiences and responses after patient safety incidents.	Tertiary
39. Lewis et al. (2013). United States.	Evidence synthesis	We report an integrative literature review of the effect of medical errors on nurses.	Integrative literature review	21 articles	Medical errors effect on nurses.	N/A
40. Lim et al. (2024). Singapore.	Original research	To explore the experience of second victim symptoms and adverse outcomes among nurses working in public healthcare institutions; understand the preferred com ponents of a structured support programme; and explore the barriers to accessing existing support strategies.	Multimethod study using quantitative survey and qualitative in‐depth interviews	12 nurses	Nurses second victim symptoms after an adverse event	Tertiary
41. Liukka et al. (2020). Finland.	Evidence synthesis	The aim is to identify the underlying elements required for damage preventing and ameliorating actions following AEs in order to provide direction for development and future investigation.	Integrative literature review	25 articles	Second victim phenomenon and actions after adverse events	N/A
42. Magaldi et al. (2021). Spain.	Original research	The aim of this work is to assess health professionals' perception of the phenomenon, as well as their capability to apply psychological first aid.	Quantitative observational descriptive study using surveys	329 participants (nurses, anaesthesiologists and anaesthesiologists in training)	Health professionals' perceptions of the second victim phenomenon	Tertiary
43. Mahat et al. (2022). Finland.	Original research	The aim of this study was to investigate negative emotions expressed by healthcare staff in their reported MAE incidents along with the immediate responses they received from their seniors and colleagues after the incident.	Retrospective study using a qualitative descriptive design and text mining	Medication administration error incidents (*n* = 72,390)	Negative emotions experienced by healthcare professionals after medication administration incidents.	N/A
44. Margulies et al. (2020). United States.	Original research	To determine the frequency of maternity health employee experiences with maternal and perinatal/neonatal adverse outcomes and gain a deeper understanding of how these experiences impact the providers.	Quantitative observational cross‐sectional study using surveys	105 participants (physicians, midwives, nurses, nurse practitioners)	Maternity health employees' experiences about perinatal or neonatal adverse outcomes and those outcomes impact to them.	Tertiary
45. Mathebula et al. (2022). South Africa.	Original research	The researchers report on the physical and psychological symptoms experienced by healthcare providers following adverse events during patient care as well as their perceptions of the quality of support received and the desired forms of support following adverse events.	Descriptive quantitative cross‐sectional study using surveys	181 healthcare providers (nurses, medical doctors and allied healthcare providers)	Psychological and physical symptoms experienced by healthcare professionals after an adverse event.	Secondary
46. McDaniel and Morris (2020). United States.	Evidence synthesis	This article reviews current knowledge about the second victim phenomenon, with a focus on the individual healthcare provider's symptoms, sequelae, stages of recovery and efforts by employers and healthcare organisations to establish a standardised process for evaluation and management of adverse and traumatic healthcare events.	Literature review	N/A	Second victim phenomenon and symptoms related to it.	N/A
47. McLaren et al. (2021). United Kingdom.	Original research	This study aimed to: review the prevalence of surgical complications experienced by ENT trainees at various stages of training, examine how such complications affected their surgical confidence and determine whether current practices allow for open discussion that enables us to learn from the incidents we are involved in.	Quantitative cross‐sectional survey	36 participants (ENT trainees)	Surgical complications impact to ENT trainees and their confidence.	N/A
48. Mira et al. (2015). Spain.	Original research	The objective of this study was to assess the effect of AEs that occur in PC and hospital settings in Spain on health professionals (second victims) in personal and professional terms.	Quantitative cross‐sectional survey	1087 health professionals	Second victims' experiences after adverse events.	Primary healthcare and hospitals
49. Mohamadi‐Bolbanabad et al. (2019). Iran.	Original research	This study aimed to investigate the prevalence of second victims, their psychosocial and physical symptoms, and its related factors among medical staff in Sanandaj hospitals in Iran.	Quantitative cross‐sectional study using surveys	338 (nurses, midwives, physicians)	Second victims psychological and physical symptoms.	Tertiary
50. Mohd Kamaruzaman et al. (2022b). Malaysia.	Original research	This study aims to investigate the factors affecting negative work‐related outcomes and resilience with a hypothetical triad of support as the mediators: colleague, supervisor and institutional support.	Quantitative cross‐sectional study using surveys	733 participants (doctors, nurses, assistant medical officers)	The effects of the second victim‐related distress.	Tertiary
51. Mohsenpour et al. (2018). Iran.	Original research	To explore the meaning of Iranian nurses' experience of ‘being a wrongdoer’.	Qualitative phenomenological study using interviews	Eight nurses	Nurses second victim experiences	General or specialist hospitals
52. Mok et al. (2020). Singapore.	Original research	The study aimed to investigate nurses' second victim experience and quality of support resources in Singapore.	Quantitative cross‐sectional study using surveys	1163 nurses	Nurses and their second victim experiences	Tertiary
53. Naya et al. (2023). Japan.	Evidence synthesis	Aim was to conduct a systematic review to explore SVS in ICU healthcare workers, including its types, prevalence, risk factors and recovery time.	Systematic review using meta‐analysis	Five studies	Second victim syndrome in healthcare workers	Tertiary
54. Neft (2023). United States.	Original research	The specific aims of this study were to identify the level of stress experienced by CRNAs at the time of a critical event and later at the time of the study, and to document the lived experiences of CRNAs who become second victims.	Multimethod study using interviews and survey.	Four participants in the qualitative part and 52 in the quantitative part (registered nurses, CRNAs)	Nurses' second victim experiences and the level of stress.	N/A
55. New and Lambeth (2024). United States.	Evidence synthesis	N/A	Literature review	N/A	Second victim phenomenon and healthcare professionals	
56. Nydoo et al. (2020). South‐Africa.	Evidence synthesis	This study aimed to explore the knowledge on the second victim phenomenon (SVP) in health care, more specifically within the speciality of obstetrics.	Literature review	13 articles	Second victim phenomenon in healthcare and specifically within obstetrics.	N/A
57. Pacutova et al. (2023). Slovakia.	Original research	The aim was to map the experiences of Slovak HCWs with PSI during the second pandemic wave, the association of these experiences with hospital management of patient safety culture (risk of recurrence, open disclosure/s victim experience) and describe their interests in receiving further training.	Quantitative cross‐sectional study using survey	193 healthcare workers	Healthcare workers experiences about patient safety incidents.	Hospital
58. Pado et al. (2023). United States.	Original research	The study aimed to test the impact of four aspects of experiencing medical mishaps on distress and posttraumatic growth.	Quantitative cross‐sectional study using survey	157 physicians and 139 nurses	Medical mishaps, distress and posttraumatic growth.	Large healthcare system
59. Pratt and Jachna (2015). Israel.	Evidence synthesis	N/A	Literature review	N/A	Clinicians second victim experiences	N/A
60. Quadros et al. (2022). Brazil.	Original research	Objective: analyse the falls of adult hospitalised patients and their repercussions on the Nursing worker as the second victim	An exploratory, descriptive and qualitative study	21 nurses and nursing technicians and 12 falls	Nurses second victim experiences after patient falls	Tertiary
61. Rinaldi et al. (2022). Italy.	Original research	We performed a study aimed at describing the prevalence of SVs among Italian nursing students, medical students and resident physicians while detecting any possible differences among such groups. As secondary objectives, we investigated the physical and psychological symptoms in the aftermath of a PSI in these groups, analysing the perceived causes and the received support.	Cross‐sectional quantitative study using survey.	387 individuals: 128 nursing students, 174 medical students and 85 residents.	Second victim prevalence and psychological and physical symptoms after patient safety incident.	N/A
62. Rivera‐Chiauzzi et al. (2022). United States.	Original research	The aim of the study was to determine the prevalence of second victim experience (SVE) among obstetrics and gynaecology (OBGYN) clinical and nonclinical healthcare workers and compare healthcare workers who did and did not identify as a second victim (SV) in the last year.	Quantitative cross‐sectional study using surveys	205 OBGYN healthcare professionals	Prevalence of second victim experience	Tertiary
63. Robertson and Long (2018). United States.	Discussion paper	The objectives of this article are to (1) discuss the impact medical error has on involved provider(s), (2) provide potential reasons why medical error can have a negative impact on provider mental health, and (3) suggest solutions for providers and healthcare organisations to recognise and mitigate the adverse effects medical error has on providers	N/A	N/A	Medical errors impact on healthcare providers	N/A
64. Ross (2018). United States.	Discussion paper	N/A	N/A	N/A	Second victims in health care	N/A
65. Sahay and McKenna (2023). Australia.	Evidence synthesis	The aim was to describe and understand what is known about nurses and nursing students as second victims.	Scoping review	23 papers	Nurses and nursing students as second victims	N/A
66. Scarpis et al. (2024). Italy.	Original research	The primary aim of our study was to determine the prevalence of HCWs involved in an adverse patient safety event in Friuli Venezia Giulia Region (Italy). The secondary aims were to use latent profile analysis to identify profiles of second victims and factors affecting the second victim and influencing profile membership, and to evaluate the relationship between the severity of symptoms and desired support options.	Quantitative cross‐sectional study using survey	733 healthcare workers, 305 included in the subsequent analyses	Healthcare workers as second victims and symptoms	Tertiary care and second level hospital
67. Schrøder and Assing Hvidt (2023). Denmark.	Original research	The aim of this study was to identify (i) emotions experienced by healthcare professionals (HCPs) after adverse or traumatic events and (ii) needs for support after adverse or traumatic events.	Qualitative descriptive study	27 seminars for a total of 198 participants (healthcare professionals)	Healthcare professionals' emotions after adverse event	Tertiary
68. Schrøder et al. (2016). Denmark.	Original research	This study investigates the self‐reported psychosocial health and well‐being of obstetricians and midwives in Denmark during the most recent 4 weeks as well as their recall of their health and well‐being immediately following their exposure to a traumatic childbirth	Quantitative cross‐sectional study using survey	1027 obstetricians and midwives	Obstetricians and midwives' health and well‐being after a traumatic event	Departments of gynaecology and obstetrics
69. Scott et al. (2009). United States.	Research article	This article describes and characterises the information obtained through this qualitative exploratory study of the experiences and recovery trajectory of past second victims.	Qualitative study using face to face interviews	31 participants (nurses, physicians and other healthcare professionals)	Experiences and recovery of second victims	N/A
70. Scott (2015). United States.	Discussion paper	N/A	N/A	N/A	Second victim experience	N/A
71. Seys et al. (2022). Belgium.	Original research	To describe the differences and similarities in the reaction of the healthcare worker involved in a patient safety incident or during the COVID‐19 pandemic.	Quantitative cross‐sectional study using survey and secondary data analysis	883 doctors and 1970 nurses	Healthcare workers' reactions to a patient safety incident	Hospital environment
72. Seys et al. (2013). Belgium.	Evidence synthesis	The objectives are to determine definitions of this concept, research the prevalence and the impact of the adverse event on the second victim, and the used coping strategies.	Systematic review	41 studies	Adverse events impact to the second victim	Healthcare setting
73. Shao et al. (2021). China.	Evidence synthesis	To explore nurses' psychological experiences after inpatient suicide.	Qualitative meta‐synthesis	11 studies	Nurses' experiences after inpatient suicide	Hospital ward
74. Shao et al. (2023). China.	Original research	The three specific aims were to (1) categorise nurses' second victim experiences into different profiles according to the support and distress they perceived; (2) describe the nurses' job insecurity in different profiles; and (3) explore nurses' turnover intention in different profiles.	Quantitative cross‐sectional descriptive study using survey	1298 nurses	Nurses' second victim experiences and distress, job insecurity and turnover intentions related to that.	Hospitals
75. Shomalinasab et al. (2023). Iran.	Original research	The present study aimed to determine the relationship between moral distress and SVS in ICUs.	Quantitative cross‐sectional study using survey, descriptive statistics and analytical tests	96 ICU nurses	Second victim syndrome and moral distress	Tertiary
76. Silveira et al. (2023). Brazil.	Original research	The aim was to understand the impacts on nursing professionals as the second victim of patient safety incidents.	Qualitative exploratory‐descriptive study	20 nursing professionals	Patient safety incidents impact on nursing professionals	Tertiary
77. Stillwater (2018). United States.	Commentary	N/A	Qualitative survey	Three school nurses	School nurses second victim experiences	Community healthcare
78. Stovall and Hansen (2021). Unites States.	Original research	To examine the relationship between nurse sociodemographic data and unique study variables with potential morally injurious outcomes (i.e., dropping out variables: changing jobs, intention to leave the profession or suicidal thinking).	Descriptive quantitative correlational study using survey	216 registered nurses	Nurses' sociodemographic data and morally injurious outcomes	N/A
79. Stovall et al. (2020). United States.	Evidence synthesis	The objective of this review of nurse second victim literature is to describe symptoms of moral injury empirically observed in nurses in the aftermath of a PSI.	Literature review	21 studies	Second victim symptoms in nurses after patient safety incident	N/A
80. Stukalin et al. (2019). Canada.	Original research	The present study is the first to use a previously validated cut‐off (≥ 24) on the ies‐r to evaluate the prevalence of clinically significant posttraumatic stress in any physician cohort after a patient ae. Our study also aimed to examine whether certain coping strategies, perceptions of the locus of controllability of the causes of the incident and perceptions of the institutional culture predicted the development of clinically significant posttraumatic stress.	Quantitative cross‐sectional study using surveys	51 participants (surgeons, medical oncologists, radiation oncologists and other specialties)	Posttraumatic stress after adverse event.	Tertiary
81. Tang et al. (2024). China.	Original research	To investigate the current status of experience and support of nurses as second victims and explore its related factors in nurses.	Multimethod study using in‐depth interviews and survey	406 nurses (survey) and 8 nurses (interview)	Nurses' second victim experience	Tertiary
82. Tartaglia and Matos (2020). Brazil.	Editorial	N/A	N/A	N/A	What is known about second victim phenomenon	N/A
83. Treiber and Jones (2018). United States.	Discussion article	This article discusses the second victim syndrome and its impacts on nurses.	N/A	N/A	Second victim syndrome on nurses	N/A
84. Ullström et al. (2014). Sweden.	Original research	The aim of this study was to investigate how healthcare professionals at a Swedish university hospital were affected by their involvement in adverse events, with emphasis on the organisational support they needed and the organisational support they received.	Qualitative study using semi‐structured interviews	21 participants (physicians, nurses, allied professionals)	Adverse events impact to healthcare professionals	Tertiary
85. Van Gerven et al. (2016a). Belgium.	Original research	This study focused on this so‐called ‘second victim’ of a patient safety incident and aimed to examine: (1) experienced symptoms in the aftermath of a patient safety incident; (2) applied coping strategies; (3) the received versus needed support and (4) the aspects that influenced whether one becomes a second victim.	Qualitative study using in‐depth interviews	31 participants (nurses, midwives, physicians)	Second victim symptoms	Hospitals
86. Van Gerven et al. (2016b). Belgium.	Original research	To examine individual, situational and organisational aspects that influence psychological impact and recovery of a patient safety incident on physicians, nurses and midwives.	Quantitative cross‐sectional study using retrospective survey	913 participants (186 physicians, 682 nurses, 45 midwives)	Patient safety incidents psychological impacts to physicians, nurses and midwives	Hospitals
87. Van Gerven et al. (2016c). Belgium.	Original research	To investigate the prevalence of healthcare professionals being personally involved in a patient safety incident (PSI), as well as the relationship of involvement and degree of harm with problematic medication use, excessive alcohol consumption, risk of burnout, work‐home interference (WHI), and turnover intentions.	Cross‐sectional quantitative survey	5788 nurses and physicians	Healthcare professionals and patient safety incidents	Tertiary
88. Vanhaecht et al. (2019). Netherlands.	Original research	To describe healthcare providers' symptoms evoked by patient safety incidents (PSIs), the duration of these symptoms and the association with the degree of patient harm caused by the incident.	Quantitative cross‐sectional study using survey	4369 (1619 doctors and 2750 nurses)	Healthcare providers' symptoms after patient safety incident	Dutch hospitals
89. Wahlberg et al. (2019). Sweden.	Original research	To explore the process that Swedish midwives and obstetricians go through after a severe event in the maternity unit.	A modified Constructivist Grounded Theory analysis, based on 14 in‐depth interviews	14 participants (midwives, obstetricians)	Midwives and obstetricians experiences after severe event.	N/A
90. Wahlberg et al. (2020). Sweden.	Original research	To explore midwives' and obstetricians' experiences, reactions and interpretations of being part of a severe event on the labour ward.	Qualitative study using in‐depth interviews	14 participants (midwives, obstetricians)	Midwives and obstetricians experiences and reactions after severe event	Tertiary
91. Wands (2021). United States.	Journal course	Upon the completion of this course, the reader should be able to: 1. Recognise the impact of second victimhood on global healthcare. 2. Describe the second victim experience. 3. Identify symptoms and anaesthesia delivery implications of the second victim experience. 4. Differentiate the stages of recovery of the second victim. 5. Formulate ideas for a departmental process for immediate support of victims.	N/A	N/A	Second victim experiences and symptoms	N/A
92. White and Delacroix (2020). United States.	Evidence synthesis	The aim of this review was to identify: how medical errors affect healthcare professionals, as second victims; and how healthcare organisations can make ‘just culture’ a reality.	Integrative review	42 studies	Medical errors impact to healthcare professionals	N/A
93. Wu et al. (2020). United States.	Evidence synthesis	This article explores terminology used to describe the professionals involved in adverse events and services to support them.	N/A	13 studies	Second victim terminology	N/A
94. Wu and Steckelberg (2012). United States.	Editorial	N/A	N/A	N/A	Medical errors and second victims	N/A
95. Yan et al. (2022). China.	Original research	Therefore, this paper aims to (a) describe the experience and support of Chinese healthcare professionals as second victims of PSIs, and (b) explore factors affecting their second victim experience and support.	Quantitative cross‐sectional study using survey	1357 Chinese healthcare professionals	Chinese healthcare professionals second victim experience	Tertiary
96. Zola et al. (2024). South‐Africa.	Original research	The emotional well‐being and clinical practice of nurses who experienced inpatient suicide at Weskoppies Psychiatric Hospital was explored.	Qualitative study using in‐depth interviews	12 nurses	Nurses' emotional well‐being after inpatient suicide	Hospital

**TABLE 4:** Number of papers and studies by country.

Country	Number of papers/studies
United States	30
Australia	5
Singapore	5
China	5
Spain	5
Iran	4
Belgium	5
Israel	3
Italy	3
Korea	3
Sweden	3
South Africa	3
Brazil	3
Austria	2
Finland	2
Ireland	2
United Kingdom	2
Netherlands	2
Canada	1
Denmark	2
Japan	1
Croatia	1
United Kingdom/United States of America	1
Chile	1
Malaysia	1
Slovakia	1
Total	96

The participants in the 28 included papers were nurses. This group encompassed registered nurses, nurse practitioners, emergency nurses, certified nurse anaesthesiologists and nursing professionals. Doctors and physicians were participants in four papers. The biggest group of participants was HCPs in 33 papers. HCPs included physicians, doctors, residents, nurses, allied HCPs, pharmacists, midwives, physician assistants, medical technicians and other allied HCPs. The study contexts primarily involved tertiary care (*n* = 35) and hospitals (*n* = 14).

### 3.2 Psychological Symptoms Healthcare Professionals Experience After a Safety Incident

Psychological symptoms related to the SVS were categorised into 10 subcategories: feeling of fear and anxiety, anger, feeling of sadness, feeling of guilt, feeling disturbed, feeling of shame, depression, self‐esteem, positive psychological symptoms and reactions and other psychological reactions and responses. These subcategories and symptoms are presented in detail in Table 5, and with the citations listed in Table 6. Only a few examples of the symptoms and impacts found are presented in the text; all the symptoms and impacts can be found in the tables.

**TABLE 5:** Psychological symptoms experienced by healthcare professionals after safety incidents.

Psychological symptoms, responses and reactions related to the second victim syndrome	Feeling of fear and anxiety (Mahat et al. 2022)	Fear Fear of errors Fear of outcomes of the errors Loss of trust Fear of lawsuits Fear of litigation Fear of patients and families Dread Scared Worry Frightened Terror Sinking feeling Horrendous Desperate Apprehension Anxiousness Existential angst Anxiety Nervous Nervousness Doubt Self‐doubt Rumination Overthinking the event Mulling Heartbroken Suffer Devastation Flashbacks and reliving Repetitive thoughts and memories Intrusive thoughts and memories Memories of what happened Distressing memories Disturbing memories Troubling memories Tangling memories Unwanted memories Terrified Terrible Horror Horrified Horrible Mortified Disbelief Dissatisfaction Awful Afraid Shock Shocking Uneasiness PTSD Acute stress reaction Acute stress disorder Posttraumatic embitterment disorder Posttraumatic stress Secondary traumatic stress Trauma Deeply traumatised Feeling traumatised Stress Stressful Cognitive stress Distress Moral distress Concern Concerns about patients' well‐being Panic Panic attack Vulnerability
	Anger (Mahat et al. 2022)	Anger Anger towards self Anger towards others Irritation Loss of temper Defensive Defensive and constructive changes Aggressive and risky behaviour Overprotective behaviour Denial
	Feeling of sadness (Mahat et al. 2022)	Sad Sadness Grief Anguish Agony Low mood Little pleasure or interest in doing things Being sorry Tearfulness Unhappy Miserable Despair Meaningless Feeling bad Disappointment Disheartened Feeling down Sorrow Unpleasant feeling Broken Negativity Pessimism
	Feeling of guilt (Mahat et al. 2022)	Guilt Feeling of judgement Feeling interrogated Weight on conscience Regret Fault Feeling of betraying Remorse Culpability Feeling humiliated “Was it my fault” Thoughts of missing something important Feeling of committing crime Wondering how they could have made such a mistake Feeling of failing Judging themselves Self‐accusation
	Feeling disturbed (Mahat et al. 2022)	Upset Agitation Over‐reaction Turmoil Disturbed Excessive excitability Feeling resentful Mood swings Obsession Hysteria
	Feeling of shame (Mahat et al. 2022)	Shame Embarrassment Self‐conscious Insufficient Inability to forgive themselves
	Depression (Mahat et al. 2022)	Depression Frustration Overwhelmed Devastation Hopeless Numbness Self‐harm Suicidal thoughts or ideation Suicidality and suicidal behaviour Suicide Feeling dejected Pain Feeling alone Demoralisation Loneliness Feeling of being on their own Loss of inner security Numb
	Self‐esteem (Mahat et al. 2022)	Loss of confidence Loss of self‐esteem and low self‐esteem Incompetent Insecurity Inadequacy Confusion Confounded Sense of failure Uncertainty Self‐hatred Questioning self‐worth Loss sense of worth Feeling stupid Lower sensation of personal accomplishment Self‐scrutiny Feeling like they were a fraud Realisation of one's insignificance Being aware of own fallibility Negative self‐evaluation
	Positive psychological symptoms and reactions	Relief Feeling determined, alert and attentive Happy that patients were not harmed Posttraumatic growth Compassion
	Other psychological reactions and responses	Hypervigilance Hyperarousal Concentration problems Alcohol and other substance use Feeling exhausted Powerlessness Spiritual and existential crise Changes in worldview Changes in personality Tension Poor memory Decreased empathy Listlessness Internal unrest Depersonalization Unable to think Emotional outburst Restlessness Feeling unreal Feeling dazed Blaming someone else Self‐blame and blame Crying Nightmares

**TABLE 6:** Psychological symptoms experienced by healthcare professionals after safety incidents with citations.

Main category	Subcategory	Symptoms from the studies
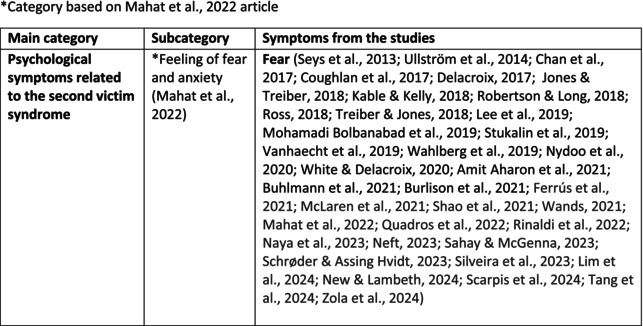		
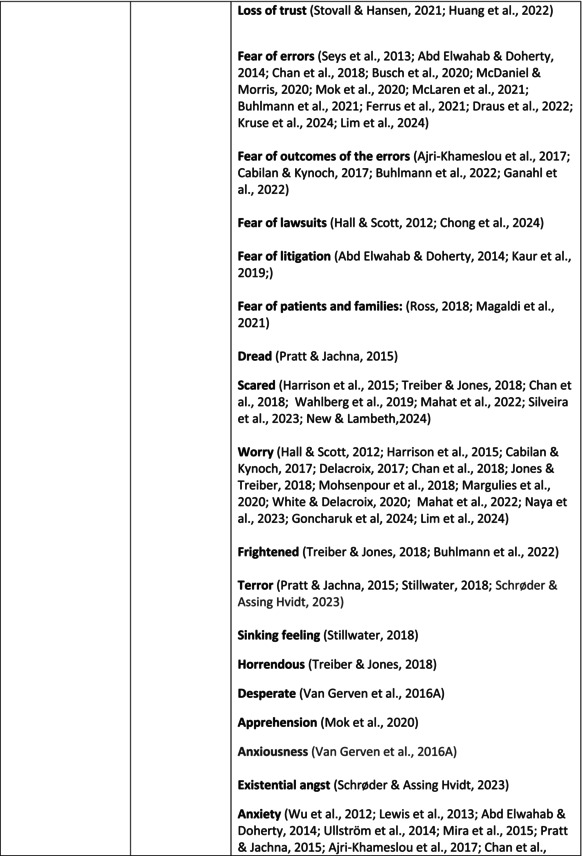		
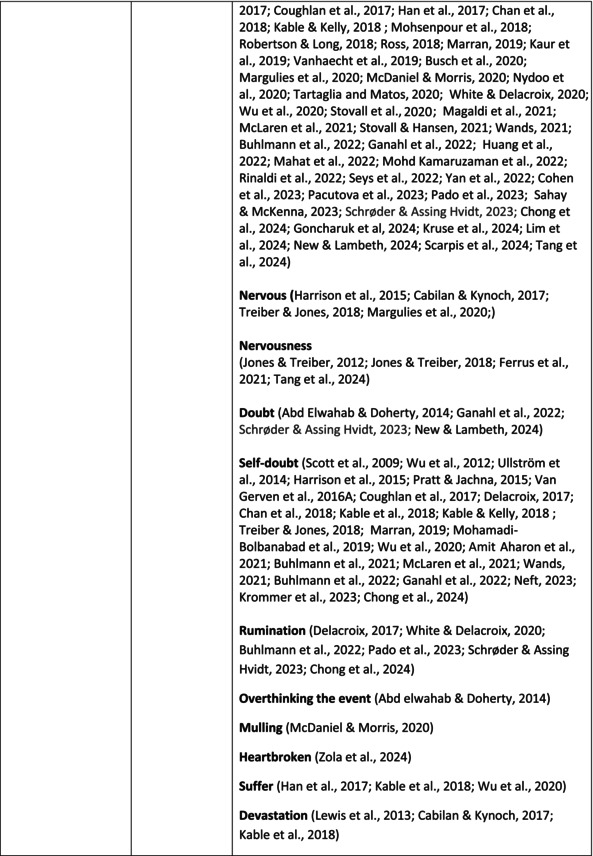		
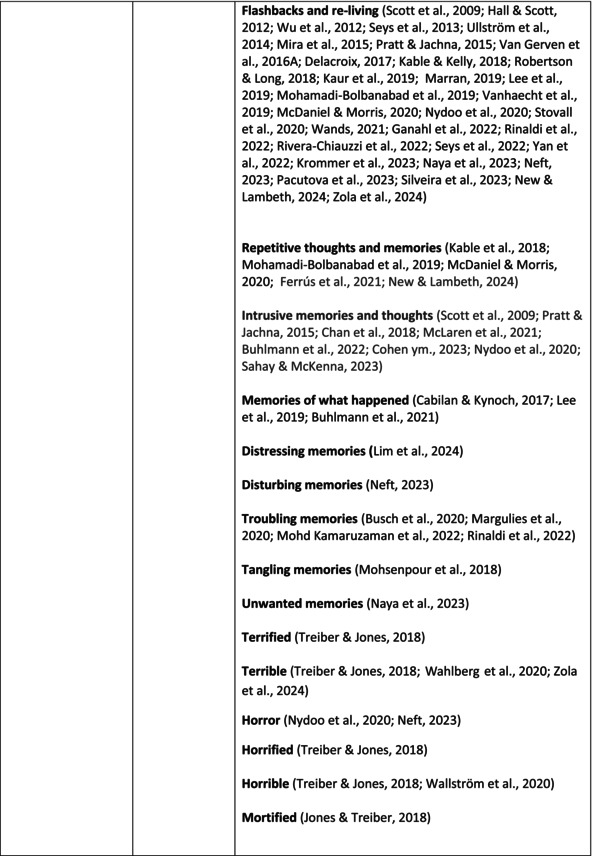		
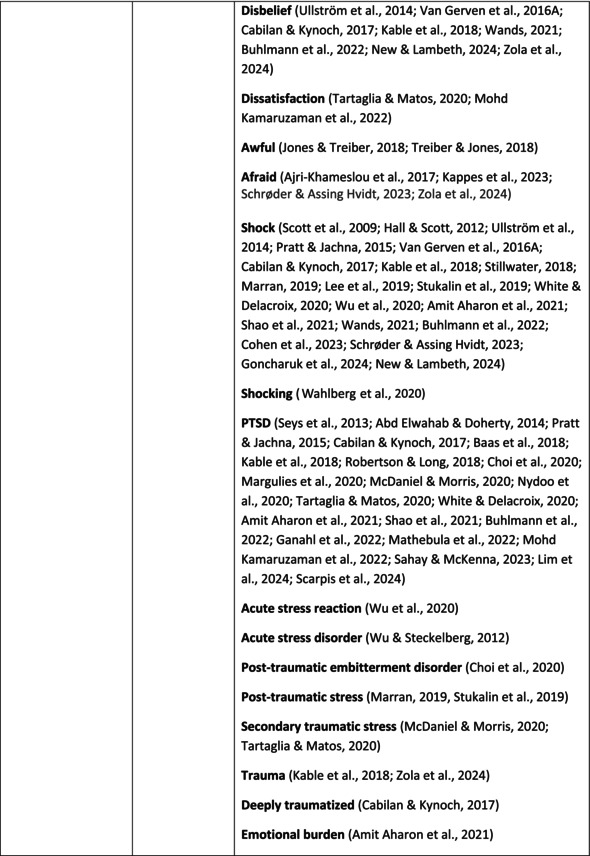		
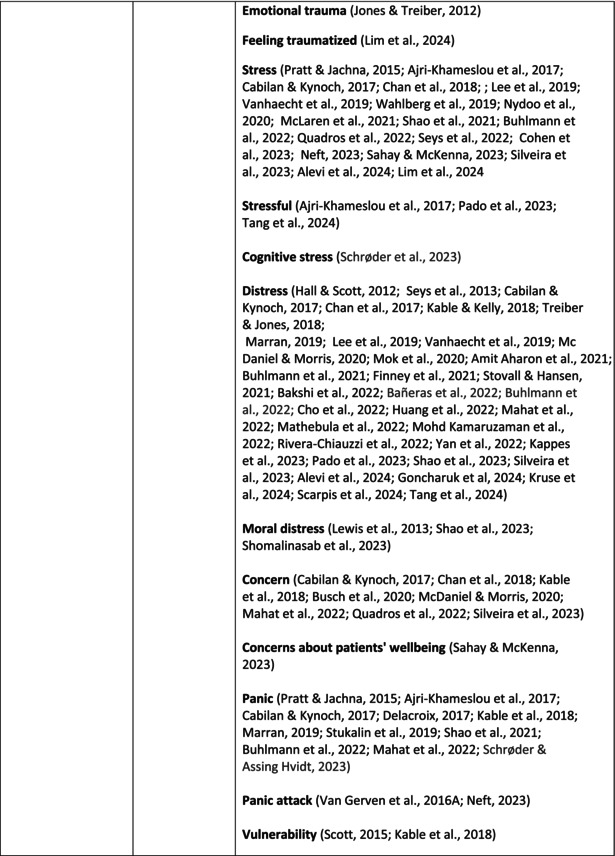		
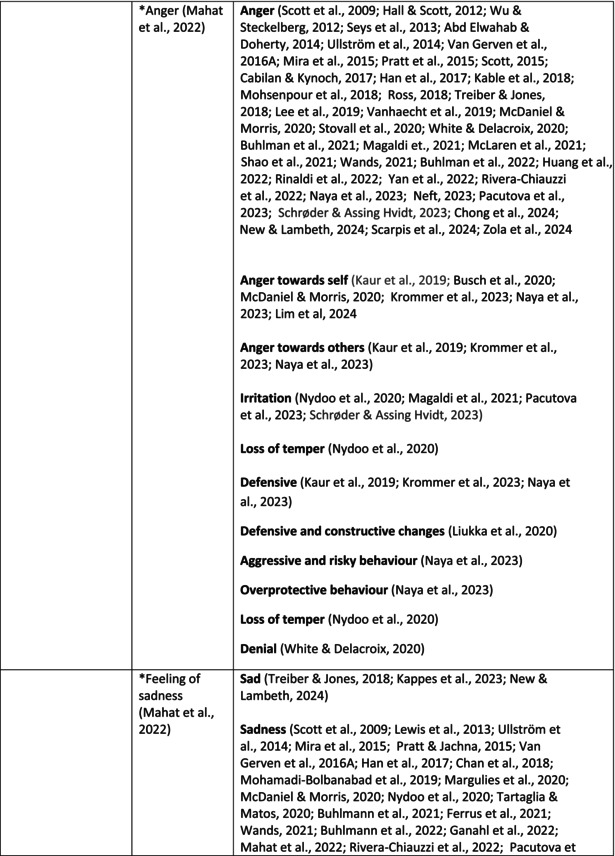		
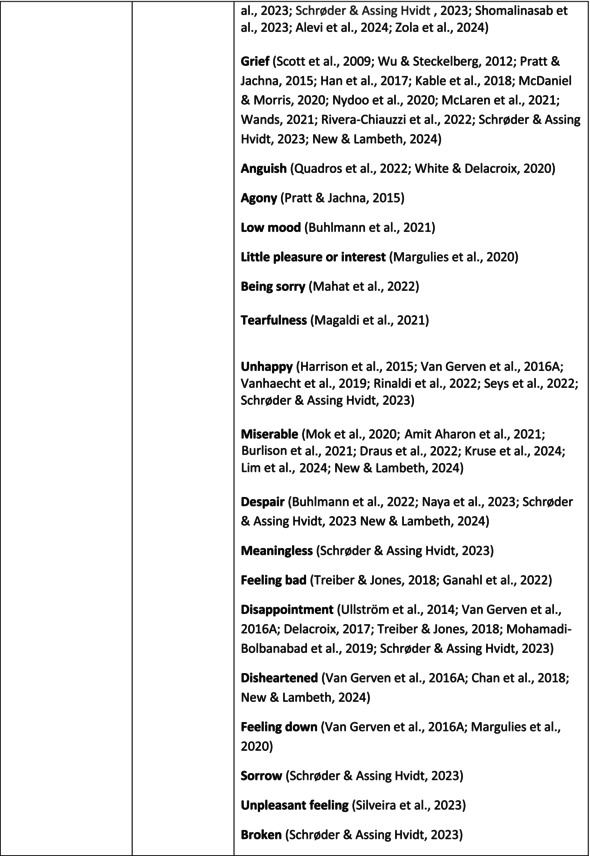		
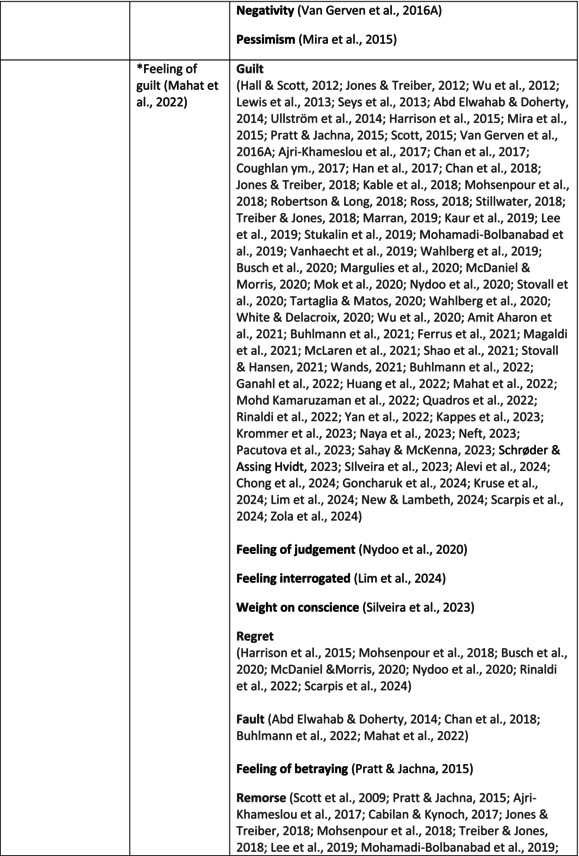		
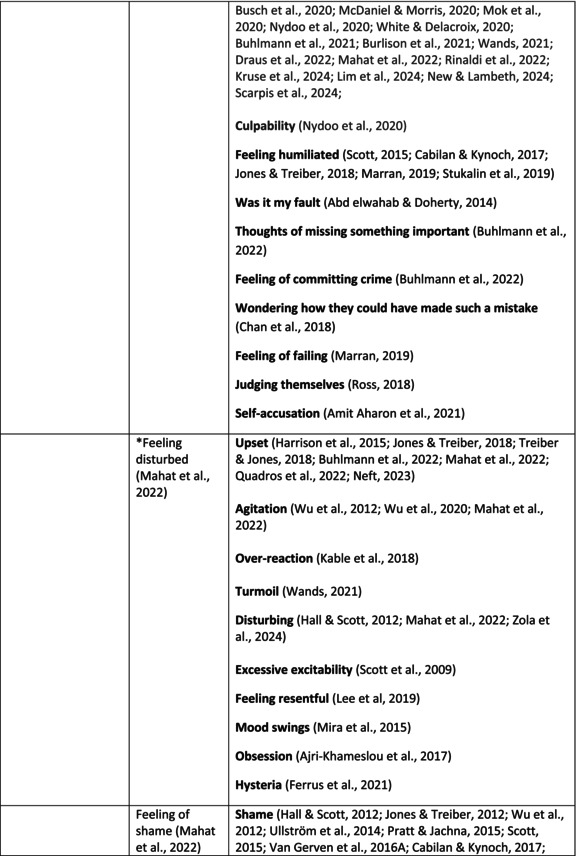		
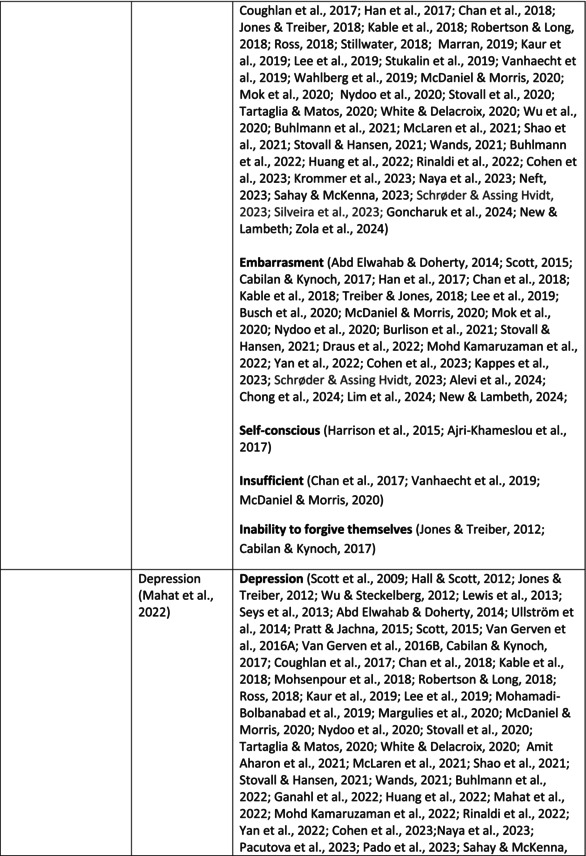		
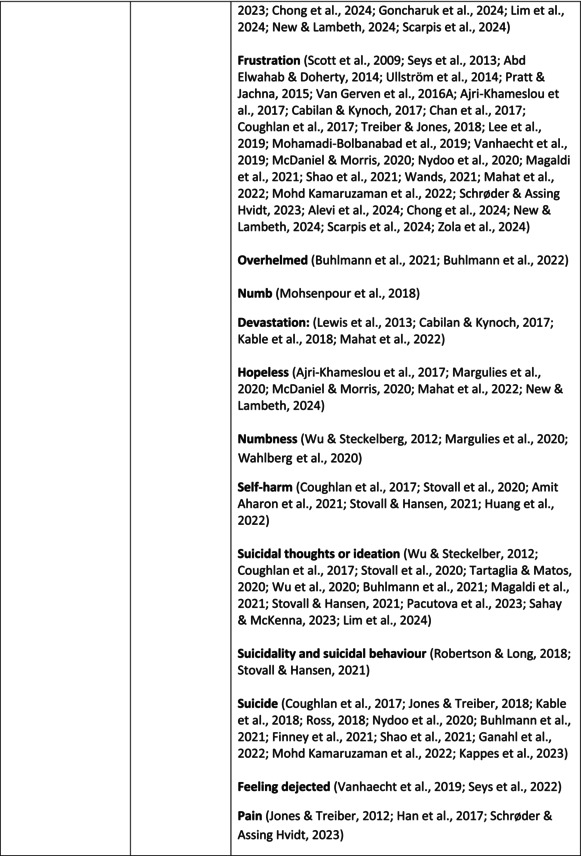		
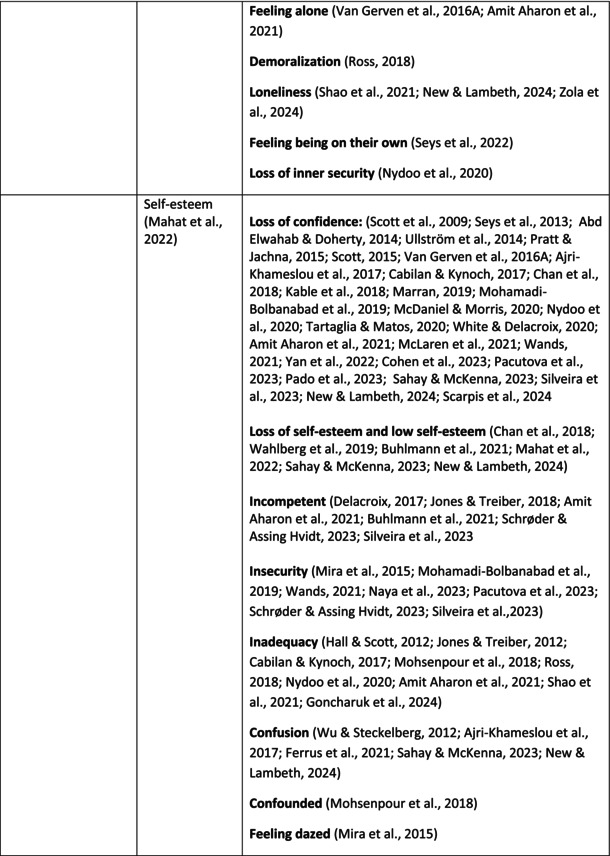		
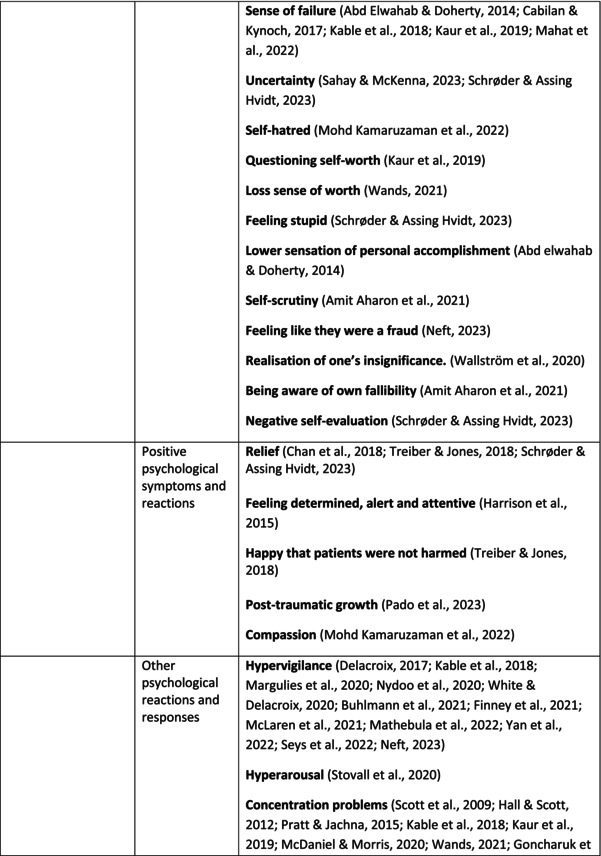		
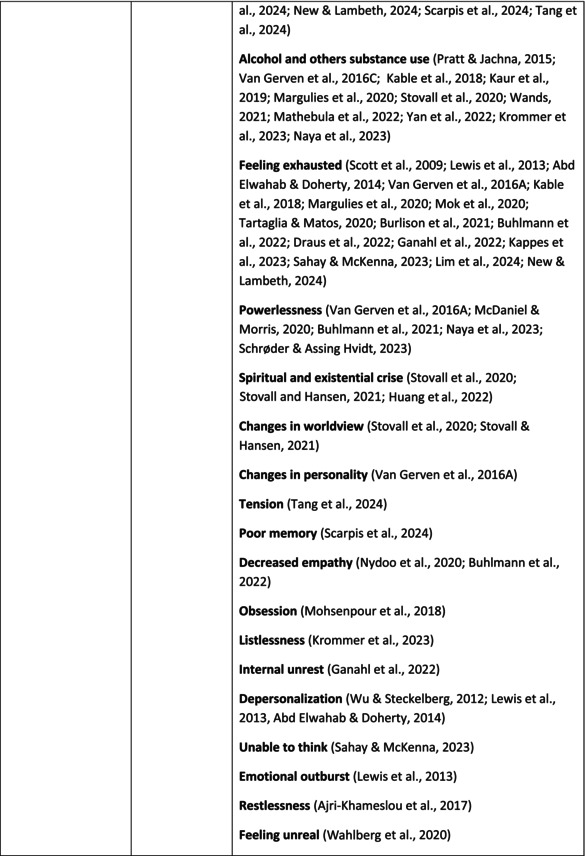		
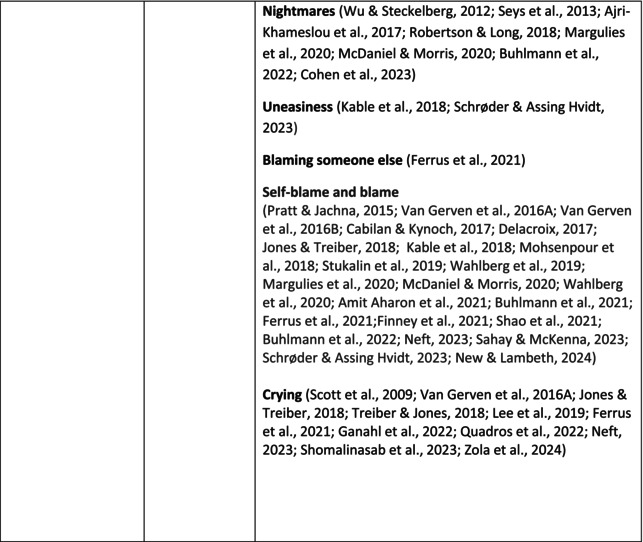		

^a^ Category based on Mahat et al. (2022) article.

HCPs experienced psychological symptoms related to fear and anxiety after the safety incidents. The most common psychological symptoms in this category were anxiety (*n* = 50) (e.g., Abd Elwahab and Doherty 2014; Mohd Kamaruzaman et al. 2022b; Pado et al. 2023) and fear (*n* = 37) (e.g., Delacroix 2017; Nydoo et al. 2020; Zola et al. 2024). Professionals also reported experiencing various emotions associated with anger, such as irritation or irritability (*n* = 4) (e.g., Magaldi et al. 2021; Pacutova et al. 2023; Schrøder and Assing Hvidt 2023) and anger towards self (*n* = 6) (e.g., Kaur et al. 2019; Krommer et al. 2023; Lim et al. 2024). The feelings of sadness were also identified in these papers. The most common emotions were sadness (*n* = 25) (e.g., Margulies et al. 2020; Rivera‐Chiauzzi et al. 2022; Schrøder and Assing Hvidt 2023) and grief (*n* = 12) (e.g., Han et al. 2017; Kable, Kelly, and Adams 2018; McDaniel and Morris 2020). Feelings of guilt were widely experienced by HCPs. Guilt (*n* = 73) (e.g., Harrison et al. 2015; Kable, Kelly, and Adams 2018; Kappes et al. 2023) was mentioned as a frequently mentioned symptom following safety incidents. Other guilt‐related symptoms included remorse (*n* = 24) (e.g., Ajri‐Khameslou et al. 2017; Cabilan and Kynoch 2017; Scarpis et al. 2024) and regret (*n* = 7) (e.g., Harrison et al. 2015; Mohsenpour et al. 2018; Scarpis et al. 2024).

HCPs felt disturbed after the incident. Psychological symptoms belonging to this category included feeling upset (*n* = 7) (e.g., Buhlmann et al. 2022; Harrison et al. 2015; Neft 2023) and agitation (Mahat et al. 2022; Wu et al. 2020; Wu and Steckelberg 2012). The feelings of shame were also common among professionals after safety incidents. The prevalent psychological symptoms found in the papers related to this category were shame (*n* = 46) (e.g., Mok et al. 2020; Naya et al. 2023; Silveira et al. 2023) and embarrassment (*n* = 24) (e.g., Abd Elwahab and Doherty 2014; Alevi et al. 2024; Chan et al. 2018). Psychological symptoms related to depression were common after safety incidents. The most common symptoms in this subcategory were depression (*n* = 50) (e.g., Mohsenpour et al. 2018; Rinaldi et al. 2022; Scarpis et al. 2024) and frustration (*n* = 27) (e.g., Lee, Pyo, Jang, Choi, and Ock 2019; Mohd Kamaruzaman et al. 2022b; Zola et al. 2024).

Safety incidents often have a significant impact on HCPs' self‐esteem. These effects were commonly described as loss of confidence (*n* = 28) (e.g., Abd Elwahab and Doherty 2014; Ajri‐Khameslou et al. 2017; Scarpis et al. 2024). Feelings of inadequacy (*n* = 9) (e.g., Goncharuk et al. 2024; Hall and Scott 2012; Mohsenpour et al. 2018) and insecurity (*n* = 7) (e.g., Mira et al. 2015; Naya et al. 2023; Silveira et al. 2023) were also associated with self‐esteem. Despite these challenges, positive psychological reactions and symptoms were also found in the included papers. HCPs reported feeling relieved (Chan et al. 2018; Schrøder and Assing Hvidt 2023; Treiber and Jones 2018) after a safety incident, for example, when patients did not experience any harm. They felt determined, alert, and attentive (Harrison et al. 2015). HCPs also felt compassion for the patients (Mohd Kamaruzaman et al. 2022b).

**FIGURE 2:**
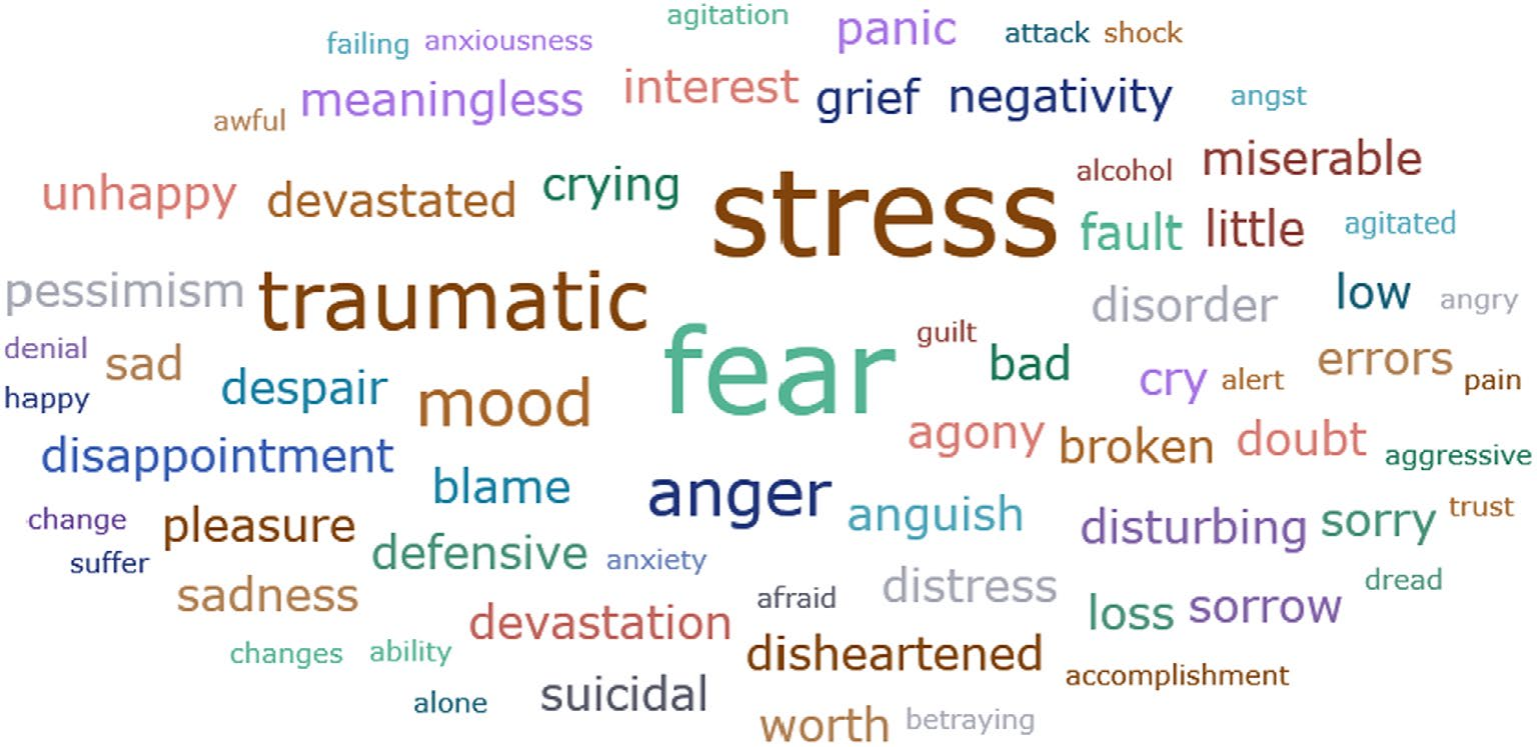
Word cloud of the most frequently occurring psychological symptoms related to the second victim syndrome.

Other psychological reactions and responses that did not fit the pre‐existing categories were grouped into their own main category and subcategories. These reactions and responses were frequently measured in the literature, such as self‐blame or blame (*n* = 23) (e.g., Delacroix 2017; Ferrús et al. 2021; Sahay and McKenna 2023), crying (*n* = 11) (e.g., Ferrús et al. 2021; Ganahl et al. 2022; Shomalinasab et al. 2023), feeling exhausted (*n* = 16) (e.g., Draus et al. 2022; Ganahl et al. 2022; Sahay and McKenna 2023), hypervigilance (*n* = 12) (e.g., Delacroix 2017; New and Lambeth 2024; Tang et al. 2024) and concentration problems (*n* = 11). The most common psychological symptoms are presented in the word cloud (Figure 2).

### 3.3 Physical Symptoms

Physical symptoms caused by SV stress were categorised into five subcategories: Symptoms related to sleep, gastrointestinal symptoms, respiratory and cardiovascular symptoms, neurological symptoms and other symptoms related to the nervous system and physical pain and tension. The results of the content analysis are presented in Table 7, and the corresponding citations listed in Table 8. Physical symptoms were defined as direct physical reactions to the incident or psychological stress professionals experienced. Symptoms related to sleep were the most commonly reported physical symptoms after a safety incident, including insomnia (*n* = 18) (e.g., Delacroix 2017; Nydoo et al. 2020; Sahay and McKenna 2023), sleep disturbance (*n* = 17) (e.g., Ullström et al. 2014; White and Delacroix 2020; Wu et al. 2020), fatigue (*n* = 17) (e.g., Coughlan et al. 2017; Kruse et al. 2024; McLaren et al. 2021) and sleeping difficulties (*n* = 12) (e.g., Abd Elwahab and Doherty 2014; Kable, Kelly, and Adams 2018; Rinaldi et al. 2022).

**TABLE 7:** Physical symptoms experienced by healthcare professionals after safety incidents.

Main category	Subcategory	Symptoms
Physical symptoms caused by second victim stress	Symptoms related to sleep	Fatigue Sleep disturbance Insomnia Sleeping difficulties Sleeping problems Loss/lack of sleep Lethargy Tiredness Low energy Hard to sleep Lack of energy Problems falling or staying in sleep Sleeping disorder Excessive need for sleep Sleep deprivation
	Gastrointestinal symptoms	Nausea/nauseous Decreased appetite Loss of appetite Eating disorder Eating difficulties Overeating Eating disturbances Indigestion Gut punch Difficult to have an appetite Change in appetite Stomach ache Stomach sickness Felt like a punch to the stomach Vomit/vomiting Abdominal pain Weight changes Losing weight Feeling sick Affected to the appetite Gastralgia Gastrointestinal discomfort
	Respiratory and cardiovascular symptoms	Rapid heart rate Racing heart Increased heart rate Triggered a heart attack Palpitations Bounding heart Tachycardia Increased blood pressure Blood pressure changes Hypertension Rapid breathing Increased respiratory rate Difficulty in breathing Dyspnoea
	Neurological symptoms and other symptoms related to the nervous system	Dizziness Queasy Tremors Loss of consciousness Hot flashes Adrenaline rush Clumsiness Shivering Feelings of being in hot or cold water Cold sweat Sweat Shaking and sweating hands Tremors in hands Exhaustion in extremities Physical distress Recoil Hot Decreased dexterity Trembling Shaking Flushed Pale Somatic stress
	Physical pain and tension	Backaches/back pain Headache Pain Muscle tension Tension Body tension

**TABLE 8:** Physical symptoms experienced by healthcare professionals after safety incidents with citations.

Main category	Subcategory	Symptoms from the studies
Physical symptoms caused by second victim stress	Symptoms related to sleep	Fatigue (Scott et al. 2009; Hall and Scott 2012; Abd Elwahab and Doherty 2014; Pratt and Jachna 2015; Scott, 2015; Cabilan and Kynoch 2017; Coughlan et al. 2017; Lee, Pyo, Jang, Choi, and Ock 2019; Mohamadi‐Bolbanabad et al. 2019; McDaniel and Morris 2020; Amit Aharon et al. 2021; McLaren et al. 2021; Wands, 2021; Ganahl et al. 2022; Schrøder and Assing Hvidt 2023; New and Lambeth 2024; Tang et al. 2024) Sleep disturbance (Scott et al. 2009; Hall and Scott 2012; Wu and Steckelberg 2012; Ullström et al. 2014; Pratt and Jachna 2015; Marran 2019; Mohamadi‐Bolbanabad et al. 2019; White and Delacroix 2020; Wu et al. 2020; Amit Aharon et al. 2021; McLaren et al. 2021; Wands, 2021; Mohd Kamaruzaman et al. 2022b; Rivera‐Chiauzzi et al. 2022; Kruse et al. 2024; Tang et al. 2024; Zola et al. 2024) Insomnia (Seys et al. 2013; Mira et al. 2015; Van Gerven et al. 2016a; Delacroix 2017; Lee, Pyo, Jang, Choi, and Ock 2019; McDaniel and Morris 2020; Nydoo et al. 2020; Buhlmann et al. 2021; Magaldi et al. 2021; Buhlmann et al. 2022; Ganahl et al. 2022; Rinaldi et al. 2022; Cohen et al. 2023; Krommer et al. 2023; Pacutova et al. 2023; Sahay and McKenna 2023; Chong et al. 2024; New and Lambeth 2024) Sleeping difficulties (Abd Elwahab and Doherty 2014; Scott 2015; Cabilan and Kynoch 2017; Chan et al. 2018; Kable, Kelly, and Adams 2018; Vanhaecht et al. 2019; McDaniel and Morris 2020; Nydoo et al. 2020; Tartaglia and Matos 2020; Rinaldi et al. 2022; Pado et al. 2023; Scarpis et al. 2024) Sleeping problems (Busch et al. 2020; Burlison et al. 2021; Kappes et al. 2023; Goncharuk et al. 2024) Loss/lack of sleep (Pratt and Jachna 2015; Kaur et al. 2019; Margulies et al. 2020; Draus et al. 2022; Naya et al. 2023) Lethargy (Mok et al. 2020) Tiredness (Mira et al. 2015; Nydoo et al. 2020; Magaldi et al. 2021; Pacutova et al. 2023; Goncharuk et al. 2024) Low energy (Goncharuk et al. 2024) Hard to sleep (Mok et al. 2020; Lim et al. 2024) Lack of energy (Schrøder and Assing Hvidt 2023) Problems falling or staying in sleep (Ganahl et al. 2022) Sleeping disorder (Schrøder et al. 2016; Choi et al. 2020; Yan et al. 2022) Excessive need for sleep (Krommer et al. 2023; Naya et al. 2023) Sleep deprivation (Seys et al. 2022)
	Gastrointestinal symptoms	Nausea/nauseous (Scott 2015; Choi et al. 2020; Mok et al. 2020; Amit Aharon et al. 2021; Buhlmann et al. 2021; Burlison et al. 2021; Ganahl et al. 2022; Mohd Kamaruzaman et al. 2022b; Cohen et al. 2023; Kappes et al. 2023; Schrøder and Assing Hvidt 2023; Shomalinasab et al. 2023; Chong et al. 2024; Lim et al. 2024; New and Lambeth 2024) Decreased appetite (New and Lambeth 2024) Loss of appetite (Van Gerven et al. 2016a; Mok et al. 2020; Burlison et al. 2021; Draus et al. 2022; Schrøder and Assing Hvidt 2023) Eating disorder (Choi et al. 2020) Eating difficulties (Sahay and McKenna 2023) Overeating (Stovall et al. 2020) Eating disturbances (Scott 2015) Indigestion (New and Lambeth 2024) Gut punch (Schrøder and Assing Hvidt 2023) Difficult to have an appetite (Mok et al. 2020; Lim et al. 2024) Change in appetite (Busch et al. 2020; Kappes et al. 2023) Stomach ache (Van Gerven et al. 2016a; Schrøder and Assing Hvidt 2023) Stomach sickness (Delacroix 2017) Felt like a punch to the stomach (Delacroix 2017) Vomit/vomiting (Van Gerven et al. 2016a; Jones and Treiber 2018) Abdominal pain (Chong et al. 2024) Weight changes (Chong et al. 2024) Losing weight (Van Gerven et al. 2016a) Feeling sick (Cabilan and Kynoch 2017; Delacroix 2017; Treiber and Jones 2018; Buhlmann et al. 2022; Draus et al. 2022; Sahay and McKenna 2023) Affected to the appetite (Kappes et al. 2023; Lim et al. 2024) Gastralgia (Mohamadi‐Bolbanabad et al. 2019) Gastrointestinal discomfort (Tang et al. 2024)
	Respiratory and cardiovascular symptoms	Rapid heart rate (Scott et al. 2009; Hall and Scott 2012; Pratt and Jachna 2015; Amit Aharon et al. 2021; Rivera‐Chiauzzi et al. 2022; Schrøder and Assing Hvidt 2023) Racing heart (Delacroix 2017; Ganahl et al. 2022; Cohen et al. 2023) Increased heart rate (Mohd Kamaruzaman et al. 2022b; New and Lambeth 2024) Triggered a heart attack (Van Gerven et al. 2016a) Palpitations (Ganahl et al. 2022; Chong et al. 2024) Bounding heart (Amit Aharon et al. 2021) Tachycardia (Mohamadi‐Bolbanabad et al. 2019; Buhlmann et al. 2021; Zola et al. 2024) Increased blood pressure (Scott et al. 2009; Hall and Scott 2012; Pratt and Jachna 2015; Amit Aharon et al. 2021; Mohd Kamaruzaman et al. 2022b; Rivera‐Chiauzzi et al. 2022) Blood pressure changes (Wands 2021) Hypertension (New and Lambeth 2024; Zola et al. 2024) Rapid breathing (Scott et al. 2009; Hall and Scott 2012; Pratt and Jachna 2015; Amit Aharon et al. 2021; New and Lambeth 2024) Increased respiratory rate (Mohd Kamaruzaman et al. 2022b) Difficulty in breathing (Delacroix 2017; Buhlmann et al. 2022) Dyspnoea (Choi et al. 2020)
	Neurological symptoms and other symptoms related to the nervous system	Dizziness (Cohen et al. 2023; Shomalinasab et al. 2023) Queasy (Lee, Pyo, Jang, Choi, and Ock 2019; Mok et al. 2020; Amit Aharon et al. 2021; Burlison et al. 2021; Ganahl et al. 2022; Kappes et al. 2023) Physical distress (Mok et al. 2020; Finney et al. 2021; Bakshi et al. 2022; Cho et al. 2022; Draus et al. 2022; Huang et al. 2022; Mathebula et al. 2022; Mohd Kamaruzaman et al. 2022b; Rivera‐Chiauzzi et al. 2022; Yan et al. 2022; Kappes et al. 2023; Shao et al. 2023; Kruse et al. 2024; Scarpis et al. 2024; Tang et al. 2024) Tremors (Lee, Pyo, Jang, Choi, and Ock 2019) Loss of consciousness (Mohsenpour et al. 2018) Hot flashes (Cohen et al. 2023; Schrøder and Assing Hvidt 2023) Adrenaline rush (Abd Elwahab and Doherty 2014) Clumsiness (Abd Elwahab and Doherty 2014) Shivering (Shao et al. 2021) Feelings of being in hot or cold water (Mohsenpour et al. 2018) Cold sweat (Choi et al. 2020; Tang et al. 2024) Sweat (Buhlmann et al. 2021; Ganahl et al. 2022; Cohen et al. 2023) Shaking and sweating hands (Van Gerven et al. 2016a) Tremors in hands (Lee, Pyo, Jang, Choi, and Ock 2019) Exhaustion in the extremities (Mohsenpour et al. 2018) Somatic stress (Schrøder and Assing Hvidt 2023) Recoil (Tang et al. 2024) Hot (Buhlmann et al. 2021) Decreased dexterity (Abd Elwahab and Doherty 2014) Trembling (Buhlmann et al. 2021) Shaking (Van Gerven et al. 2016a; Buhlmann et al. 2021) Flushed (Delacroix 2017) Pale (Mohsenpour et al. 2018)
	Physical pain and tension	Backaches/back pain (Krommer et al. 2023; Naya et al. 2023) Headache (Scott 2015; Lee, Pyo, Jang, Choi, and Ock 2019; Mohamadi‐Bolbanabad et al. 2019; Cohen et al. 2023; Krommer et al. 2023; Naya et al. 2023; Chong et al. 2024; New and Lambeth 2024) Pain (Lee, Pyo, Jang, Choi, and Ock 2019; Tang et al. 2024) Muscle tension (Scott et al. 2009; Hall and Scott 2012; Pratt and Jachna 2015; Scott 2015; Mohamadi‐Bolbanabad et al. 2019; New and Lambeth 2024) Tension (Wands 2021) Body tension (Choi et al. 2020)

HCPs experienced gastrointestinal symptoms after a safety incident, such as nausea (*n* = 15) (e.g., Choi et al. 2020; Mohd Kamaruzaman et al. 2022a; Shomalinasab et al. 2023) and loss of appetite (*n* = 5) (e.g., Burlison et al. 2021; Draus et al. 2022; Schrøder and Assing Hvidt 2023). Less common gastrointestinal symptoms reported in the papers were symptoms like indigestion (New and Lambeth 2024). Respiratory and cardiovascular symptoms included increased blood pressure (*n* = 6) (e.g., Hall and Scott 2012; Mohd Kamaruzaman et al. 2022a; Rivera‐Chiauzzi et al. 2022), rapid heart rate (*n* = 6) (e.g., Hall and Scott 2012; Pratt and Jachna 2015; Rivera‐Chiauzzi et al. 2022) and rapid breathing (*n* = 5) (e.g., Amit Aharon et al. 2021; New and Lambeth 2024). Dyspnoea was an uncommon respiratory‐related symptom (Choi et al. 2020).

**FIGURE 3:**
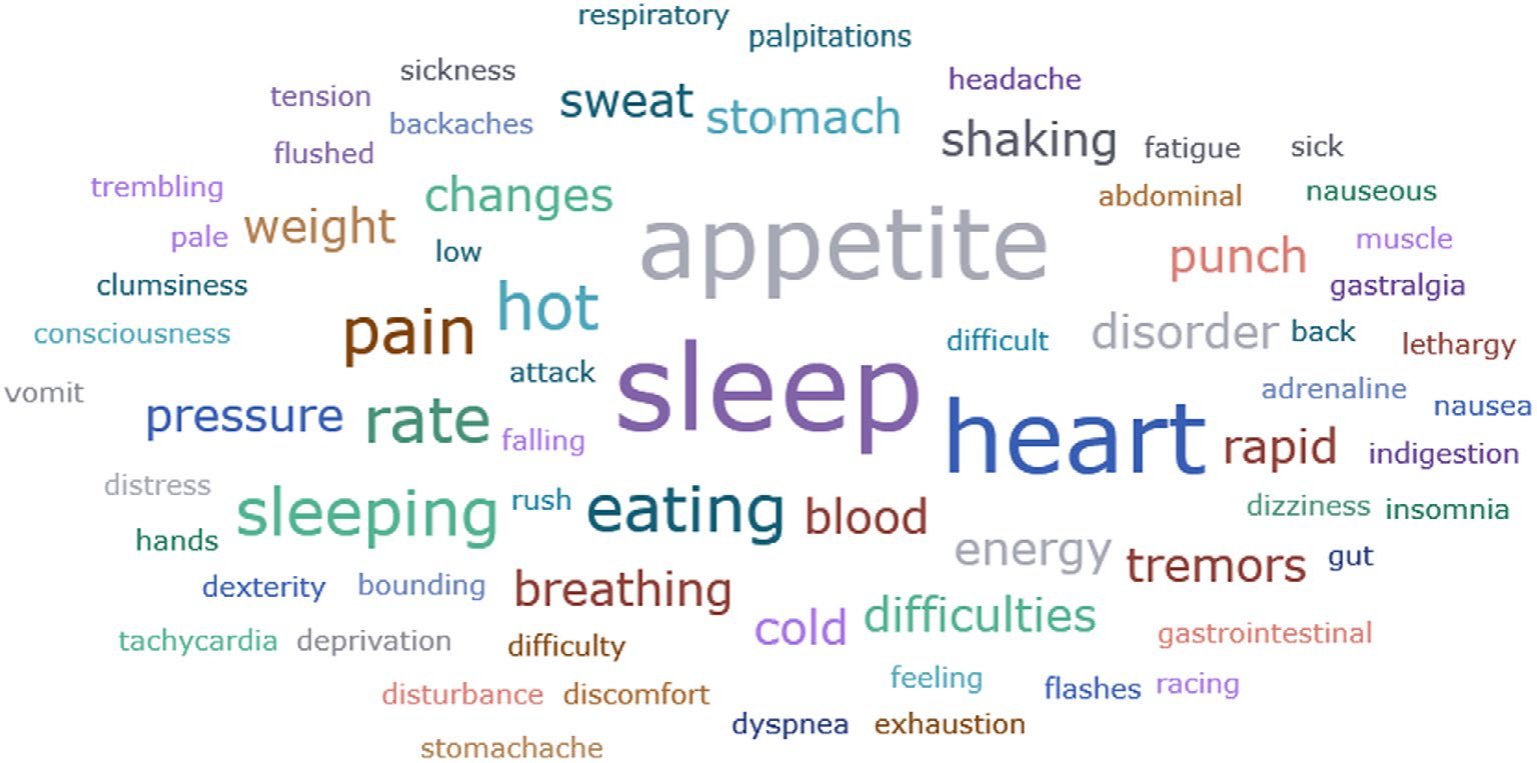
Word cloud of the most frequently occurring physical symptoms related to the second victim syndrome.

Neurological symptoms and symptoms related to the nervous system were such as feeling queasy (*n* = 6) (e.g., Amit Aharon et al. 2021; Burlison et al. 2021; Kappes et al. 2023), sweating (Buhlmann et al. 2021; Ganahl et al. 2022; Cohen et al. 2023) and shaking (Buhlmann et al. 2021; Van Gerven et al. 2016). HCPs experienced physical distress, tension and pain. Headache (*n* = 7) (e.g., Cohen et al. 2023; Krommer et al. 2023; Naya et al. 2023) was the most common pain experienced after a safety incident. Muscle tension (*n* = 6) (e.g., Hall and Scott 2012; New and Lambeth 2024; Pratt and Jachna 2015) and backaches or back pain (Krommer et al. 2023; Naya et al. 2023) were other possible symptoms that HCPs experienced. The most prevalent physical symptoms were visually represented in the word cloud (Figure 3).

### 3.4 Professional and Social Impact

Patient safety incidents had an impact on the professional and social lives of HCPs. The impacts found were categorised into 17 categories: Reduced professional self‐efficacy, professional self‐doubt, turnover intentions, absenteeism, burnout, decreased job satisfaction, fear of risk of future errors, job security stress, mistrust in colleagues, long‐lasting professional grief, learning from errors, becoming more alert at work, valued colleague relationships, social withdrawal, social discrimination and negative emotions related to sociality. HCPs experienced reduced professional self‐efficacy, characterised by feelings of inadequacy, lack of concentration at work, reduced confidence, sub‐optimal performance, reduced cognitive functioning, low motivation, impaired decision‐making and clinical judgement ability and frequent distraction from work (*n* = 37 studies) (e.g., Ajri‐Khameslou et al. 2017; Burlison et al. 2021; Cho et al. 2022). HCPs also showed professional self‐doubt regarding their work abilities and competencies (*n* = 39) (e.g., Delacroix 2017; Finney et al. 2021; Sahay and McKenna 2023). They were found to avoid or abstain from patient care, often questioned their clinical skills and competency, second‐guessed their career choice as HCPs and emotionally detached themselves from the patients and patient care activities (e.g., Kable, Kelly, and Adams 2018; Mohamadi‐Bolbanabad et al. 2019; Zola et al. 2024).

Numerous studies (*n* = 43) showed that HCPs expressed turnover intentions after becoming involved in patient safety incidents (e.g., Burlison et al. 2021; Finney et al. 2021; Lim et al. 2024). Some considered changing careers, others wanted to leave the department where the incident occurred, some wanted to change the workplace, and some expressed their desire to resign and quit their jobs (e.g., Coughlan et al. 2017; Amit Aharon et al. 2021; Kruse et al. 2024). Although HCPs in the included studies demonstrated a greater intention to turnover, work absenteeism was observed in relatively few studies (*n* = 17) (e.g., Amit Aharon et al. 2021; Bakshi et al. 2022; Kappes et al. 2023). Burnout (*n* = 21) was another prevalent outcome after a safety incident (e.g., Cabilan and Kynoch 2017; Ganahl et al. 2022; Shomalinasab et al. 2023).

HCPs also experienced decreased job satisfaction (*n* = 22) (e.g., Ajri‐Khameslou et al. 2017; Mathebula et al. 2022; Zola et al. 2024). They faced a fear of the risk of future errors (*n* = 16) while performing their work (e.g., Buhlmann et al. 2021; Neft 2023; Scarpis et al. 2024). They experienced job security stress (*n* = 24), expressed as a fear of job loss (e.g., Chan et al. 2018; Krommer et al. 2023; Naya et al. 2023). Mistrust in colleagues (*n* = 27) was another professional impact after a safety incident (e.g., Cabilan and Kynoch 2017; Krommer et al. 2023; Lim et al. 2024). Long‐lasting professional grief (*n* = 12) was also reported (e.g., Marran 2019; Quadros et al. 2022; New and Lambeth 2024).

Regarding the social impact of patient safety incidents, HCPs opted to change the location of jobs and residences (*n* = 8) (e.g., Bakshi et al. 2022; Kable, Kelly, and Adams 2018; Shao et al. 2021). They faced difficulties in their interpersonal relationships (Margulies et al. 2020) and experienced social discrimination from colleagues (Chan et al. 2018; Sahay and McKenna 2023; New and Lambeth 2024). Two papers reported social withdrawal symptoms (Stovall et al. 2020; Stovall and Hansen 2021). HCPs were also found to have negative emotions related to sociality, such as an inferiority complex, emptiness and loneliness (Scott et al. 2009; Schrøder and Assing Hvidt 2023).

#### 3.4.1 Positive Impacts After Safety Incidents

In addition to this negative impact, some studies found a positive impact of patient safety incidents among HCPs. HCPs mentioned learning valuable lessons after being involved in patient safety incidents (*n* = 15) (e.g., Liukka et al. 2020; Buhlmann et al. 2022; Silveira et al. 2023). Some of them took the error as a catalyst for change, while others took it as an inspiration for improvement (Buhlmann et al. 2021). Some HCPs viewed it as a learning experience (e.g., Liukka et al. 2020; Nydoo et al. 2020; Pado et al. 2023) and even said that they were willing to learn more (Coughlan et al. 2017), grow as professionals (Buhlmann et al. 2021; Shao et al. 2021; Pado et al. 2023) and make positive changes (Robertson and Long 2018) after their involvement in patient safety incidents. HCPs reported becoming more alert at work (*n* = 12) (e.g., Ganahl et al. 2022; Pacutova et al. 2023; Tang et al. 2024). Some also felt more focused, and the incident enhanced their skills and assertiveness. They also reported being more confident when seeking information and consulting (e.g., Pado et al. 2023; Silveira et al. 2023; Zola et al. 2024). HCPs who received support after being involved in patient safety incidents were keen to support colleagues facing similar situations. They valued relationships with their colleagues (*n* = 4). When HCPs felt supported after a safety incident, their relationships and communications with their colleagues also improved. They were also able to direct their colleagues towards the support strategies in similar situations (Lewis et al. 2013; Harrison et al. 2015; Nydoo et al. 2020).

**FIGURE 4:**
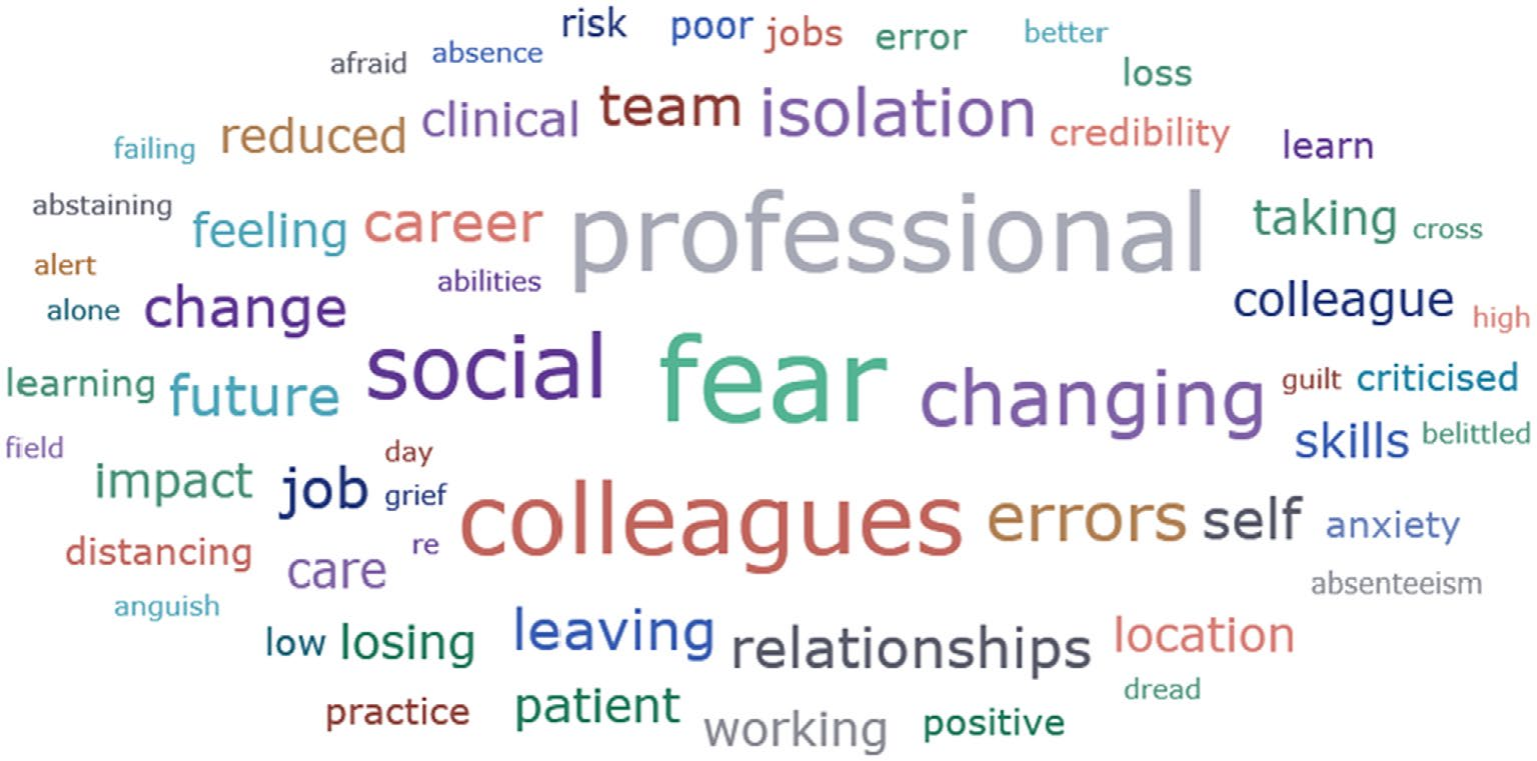
Word cloud of the most frequently occurring professional and social impacts after a safety incident.

The most common professional and social impacts on HCPs are visually presented in the word cloud (Figure 4). The results of the synthesis are presented in detail with citations in Table 9.

**TABLE 9:** Professional and social impacts experienced by healthcare professionals after safety incidents.

Authors and date	Main category	Codes related to the category
Abd Elwahab and Doherty (2014), Ajri‐Khameslou et al. (2017), Amit Aharon et al. (2021), Burlison et al. (2021), Cho et al. (2022), Cohen et al. (2023), Finney et al. (2021), Huang et al. (2022), Jones and Treiber (2012), Kruse et al. (2024), Magaldi et al. (2021), Mathebula et al. (2022), Lee, Pyo, Jang, Choi, and Ock (2019), Lewis et al. (2013), Lim et al. (2024), Zola et al. (2024), Yan et al. (2022), Wu and Steckelberg (2012), White and Delacroix (2020), Wands (2021), Vanhaecht et al. (2019), Van Gerven et al. (2016) Increased risk of …], Van Gerven et al. (2016) [Personal, situational and organisational…], Ullström et al. (2014), Treiber and Jones (2018), Tartaglia and Matos (2020), Tang et al. (2024), Stillwater (2018), Silveira et al. (2023), Mok et al. (2020), Nydoo et al. (2020), Pratt and Jachna (2015), Rivera‐Chiauzzi et al. (2022), Robertson and Long (2018), Scarpis et al. (2024), Schrøder and Assing Hvidt (2023), Scott et al. (2009), Scott et al. (2015)	Reduced professional self‐efficacy	Improper decision‐making Impaired clinical judgement Reduced capability/workability Low motivation Low professional self‐esteem Feeling of inadequacy regarding patient care abilities/workability Difficulty/lack of concentration at work Reduced cognitive functioning Loss of professional confidence Difficulty performing medical practices Poor concentration at work Poor memory Sub‐optimal performance Unable to provide quality care Distraction at work
Alevi et al. (2024); Amit Aharon et al. (2021), Buhlmann et al. (2022), Cabilan and Kynoch (2017), Chong et al. (2024), Delacroix (2017), Finney et al. (2021), Ganahl et al. (2022), Hall and Scott (2012); Jones and Treiber (2018); Kable, Kelly, and Adams (2018); Kappes et al. (2023); Kaur et al. (2019); Mahat et al. (2022); Margulies et al. (2020), Lewis et al. (2013), Lim et al. (2024), Liukka et al. (2020), Zola et al. (2024), Yan et al. (2022), Wu and Steckelberg (2012), Vanhaecht et al. (2019), Van Gerven et al. (2016) [Personal, situational and organisational…], Ullström et al. (2014), Treiber and Jones (2018), Tang et al. (2024), Silveira et al. (2023), Mok et al. (2020), Naya et al. (2023), Neft (2023), New and Lambeth (2024), Pratt and Jachna (2015), Quadros et al. (2022), Rinaldi et al. (2022), Robertson and Long (2018), Sahay and McKenna (2023), Schrøder and Assing Hvidt (2023), Scott et al. (2009), Scott et al. (2015), Seys et al. (2022), Shao et al. (2021)	Professional self‐doubt	Avoidance of patient care Emotional distancing/detachment Avoiding or abstaining from doing certain procedures Inability and insecurity to perform their duties at work Questioning their own skills Second guessing the career Struggling alone in isolation Distancing from patients Anxiety about work Questioning professional competency
Amit Aharon et al. (2021); Bakshi et al. (2022); Bañeras et al. (2022); Buhlmann et al. (2022)¸Burlison et al. (2021)¸Cho et al. (2022); Cho et al. (2022); Cohen et al. (2023): Finney et al. (2021); Ganahl et al. (2022); Huang et al. (2022); Kappes et al. (2023); Kaur et al. (2019); Kruse et al. (2024); Lewis et al. (2013); Lim et al. (2024); Magaldi et al. (2021); Mathebula et al. (2022), Lee, Pyo, Jang, Choi, and Ock (2019), Lewis et al. (2013), Lim et al. (2024), Wu and Steckelberg (2012), Wu et al. (2020), Wands (2021), Vanhaecht et al. (2019), Van Gerven et al. (2016) [Increased risk of …], Van Gerven et al. (2016) [Personal, situational and organisational…], Stovall et al. (2020), Stovall and Hansen (2021), Mok et al. (2020), New and Lambeth (2024), Nydoo et al. (2020), Pratt and Jachna (2015), Rivera‐Chiauzzi et al. (2022), Robertson and Long (2018), Sahay and McKenna (2023), Scarpis et al. (2024), Schrøder and Assing Hvidt (2023), Schrøder et al. (2016), Scott et al. (2009), Scott et al. (2015), Seys et al. (2022), Shao et al. (2021), Shao et al. (2023)	Turnover intentions	Leaving the department or profession Changing jobs Changing the direction of career Leaving the clinical setting Leaving the career Intention to drop out Giving up Changing the location Quitting Consideration for career change, or resignation Change vocations Changing the workplace Leaving the field Switch jobs
Amit Aharon et al. (2021)¸ Bakshi et al. (2022); Bañeras et al. (2022)¸Burlison et al. (2021)¸Cho et al. (2022); Finney et al. (2021); Huang et al. (2022); Kappes et al. (2023); Kruse et al. (2024); Mathebula et al. (2022), Yan et al. (2022), Ullström et al. (2014), Mok et al. (2020), New and Lambeth (2024), Rivera‐Chiauzzi et al. (2022), Sahay and McKenna (2023), Scarpis et al. (2024)	Absenteeism	Taking absence from work Taking time‐off Taking the day off Sick leave
Cabilan and Kynoch (2017); Marran (2019); Ganahl et al. (2022), Lewis et al. (2013), Yan et al. (2022), Vanhaecht et al. (2019), Van Gerven et al. (2016) Increased risk of …], Van Gerven et al. (2016) [Personal, situational and organisational…], Ullström et al. (2014), Treiber and Jones (2018), Stovall and Hansen (2021), New and Lambeth (2024), Nydoo et al. (2020), Pacutova et al. (2023), Pado et al. (2023), Ross (2018), Scarpis et al. (2024), Schrøder et al. (2016), Seys et al. (2013), Shao et al. (2021), Shomalinasab et al. (2023)	Burnout	Burnout
McDaniel and Morris (2020), Shomalinasab et al. (2023), Zola et al. (2024), Abd Elwahab and Doherty (2014), Ajri‐Khameslou et al. (2017), Buhlmann et al. (2022), Chong et al. (2024), Kruse et al. (2024), Lee, Pyo, Jang, Choi, and Ock (2019), Lewis et al. (2013), Mathebula et al. (2022), McLaren et al. (2021), Mohamadi‐Bolbanabad et al. (2019), Nydoo et al. (2020), Pado et al. (2023), Pratt and Jachna (2015), Scarpis et al. (2024), Scott et al. (2009), Shao et al. (2021), Tartaglia and Matos (2020), Ullström et al. (2014), White and Delacroix (2020)	Decreased job satisfaction	
Buhlmann et al. (2021); Margulies et al. (2020), Zola et al. (2024), Vanhaecht et al. (2019), Van Gerven et al. (2016) Increased risk of …], Tang et al. (2024), Silveira et al. (2023), Mok et al. (2020), Neft (2023), Pacutova et al. (2023), Rinaldi et al. (2022), Ross (2018), Sahay and McKenna (2023), Scarpis et al. (2024), Schrøder and Assing Hvidt (2023), Shao et al. (2021)	Fear of risk of future errors	Dread of making another error Hypervigilance at work Multiple checks Constantly on guard for errors Afraid of performing high‐risk procedures Overly attentive
Buhlmann et al. (2021); Cabilan and Kynoch (2017), Chan et al. (2018); Delacroix (2017); Ferrús et al. (2021); Hall and Scott (2012); Kable, Kelly, and Adams (2018), Krommer et al. (2023), White and Delacroix (2020), Wands (2021), Van Gerven et al. (2016) [Personal, situational and organisational…], Ullström et al. (2014), Treiber and Jones (2018), Naya et al. (2023), Neft (2023), New and Lambeth (2024), Nydoo et al. (2020), Pado et al. (2023), Pratt and Jachna (2015), Quadros et al. (2022), Ross (2018), Sahay and McKenna (2023), Scott et al. (2009), Shao et al. (2021), Shao et al. (2023)	Job security stress	Worry about job Loss of reputation Uncertain future Fear of dismissal Fear of criminal indictment Fear of punishment Fear of losing professional credibility Revoke of licence Issues of clinical credibility, respect Contemplating work future Fear of losing job Fear of litigation
Cabilan and Kynoch (2017)¸Chan et al. (2018); Harrison et al. (2015); Jones and Treiber, (2018); Kable, Kelly, and Adams (2018); Kaur et al. (2019); Krommer et al. (2023); Lee, Pyo, Jang, Choi, and Ock (2019); Lim et al. (2024); Liukka et al. (2020), Lee, Pyo, Jang, Choi, and Ock (2019), Lim et al. (2024), Zola et al. (2024), Wands (2021), Wahlberg et al. (2020), Treiber and Jones (2018), Tang et al. (2024), Naya et al. (2023), New and Lambeth (2024), Pratt and Jachna (2015), Quadros et al. (2022), Rinaldi et al. (2022), Sahay and McKenna (2023), Schrøder and Assing Hvidt (2023), Seys et al. (2022), Seys et al. (2013)	Mistrust in colleagues	Less motivation to work in team Exhausted working in a team Disrespect from colleagues Mocked for errors Being black marked Gossiping Belittled by colleagues Fear of rejection Strained colleague relationships Professional isolation Fear of judgement Fear of exclusion by colleagues Publicly criticised after an error Negative impact on workplace relations Struggle to keep working in the same unit Criticised and censured for mistake Uneasiness in team working Losing the trust of colleagues and manager Being blamed Feeling uncertain in team
Cohen et al. (2023); Marran (2019); Ganahl et al. (2022); Kable, Kelly, and Adams (2018).; Krommer et al. (2023), Lewis et al. (2013), Tang et al. (2024), New and Lambeth (2024), Pratt and Jachna (2015), Rinaldi et al. (2022), Quadros et al. (2022), Ross (2018), Schrøder and Assing Hvidt (2023), Scott et al. (2009).	Long‐lasting professional grief	Severe anxiety about returning to work Challenges in maintaining a healthy work–life balance Feeling personally responsible for errors Failing the patient Never recovering from the guilt Reliving the situation in similar circumstances at work Denial (trying to forget that the event occurred) Practice distress Decreased meaning of work Professional anguish Change in work behaviour Experiencing stigma
Positive impact		
Buhlmann et al. (2022); Buhlmann et al. (2021); Cohen et al. (2023); Lewis et al. (2013), Lewis et al. (2013), Liukka et al. (2020), Van Gerven et al. (2016) [Personal, situational and organisational…], Silveira et al. (2023), Nydoo et al. (2020), Pado et al. (2023), Robertson and Long (2018), Schrøder and Assing Hvidt (2023), Shao et al. (2021)	Learning from errors	Learning from experiences Valuable lessons Catalyst for change Inspiration for improvement Willingness to learn Motivation to improve Reflecting practice and grow as a person Attempting to make positive changes
Buhlmann et al. (2022); Cohen et al. (2023); Ganahl et al. (2022), Lewis et al. (2013), Zola et al. (2024), Wu et al. (2020), Van Gerven et al. (2016) [Personal, situational and organisational…], Tang et al. (2024), Silveira et al. (2023), Nydoo et al. (2020), Pacutova et al. (2023), Pado et al. (2023)	Becoming more alert at work	Cautious in future work Enhancing skills Increasing attention Seeking information and consultation Heightened awareness Vigilance Increased assertiveness More focused on work Being more assertive Extra carefulness Cross‐checking Improving skills Willingness to learn
Harrison et al. (2015), Lewis et al. (2013), Nydoo et al. (2020), Pado et al. (2023)	Valued colleague relationships	Improved colleague relationships when felt supported Supporting other colleagues in need Better communication with colleagues
Social Impact		
Kable, Kelly, and Adams (2018), Krommer et al. (2023), Margulies et al. (2020), White and Delacroix (2020), Nydoo et al. (2020), Pratt and Jachna (2015), Scott et al. (2009), Scott et al. (2015)	Changing the location	Changing the location/residence Social isolation Difficulties in interpersonal relationships Isolation Social discrimination by colleagues Relocating
Stovall et al. (2020), Stovall and Hansen (2021)	Social withdrawal	Social withdrawal Avoidance Social problems
New and Lambeth (2024), Sahay and McKenna (2023)	Social discrimination	
Schrøder and Assing Hvidt (2023), Scott et al. (2009)	Negative emotions related to sociality	Loneliness Vulnerability Emptiness Inferiority Self‐isolation

## 4 Discussion

This scoping review described the types of psychological and physical symptoms experienced by HCPs who became SVs after a patient safety incident and outlines the impact it had on their social and professional lives. The contexts where the included papers were conducted included community health and primary, secondary and tertiary care units. In this scoping review, the symptoms and impacts after a safety incident were examined by all professional groups and in various healthcare contexts. This study explored a broad range of concepts related to the SVS from 96 papers, which facilitated mapping of the prevalence of specified experienced symptoms documented in the literature. The well‐being of HCPs is essential for the effective functioning of healthcare systems. While safety incidents cannot be entirely avoided in healthcare, it is necessary to understand and map their consequences to professionals.

Furthermore, this scoping review highlighted the often‐present but easily overlooked positive impacts that patient safety incidents might have on the professional and social lives of involved HCPs, such as learning from errors, increased attentiveness at work and valuing collegial relationships. Previous studies have found that learning from experience is one of the most effective forms of learning, as it provides HCPs with an opportunity to reflect on the event and identify ways to prevent the recurrence of similar incidents (Wu et al. 1991; Palominos et al. 2019; Gemmete 2024). The importance of learning from mistakes has been confirmed by prior literature; in addition to improving the quality culture within healthcare organisations, learning from mistakes also contributes to the professional growth and healing process of HCPs affected by adverse events (Chan et al. 2017).

It is natural and an inherent part of the profession that HCPs experience a wide range of emotions while working in patient care. Although these emotions are a normal aspect of the job, they can have both negative and positive impacts on professionals. (Sattar et al. 2024) SVS‐related emotions have been widely studied, but a comprehensive overview of all symptoms and impacts related to social and professional life has not been conducted. Anger, fear and loss of emotions have been identified as typical emotions related to the SVS (Bushuven et al. 2022). This scoping review confirmed that anger and sadness are common psychological symptoms after a safety incident and that these terms encompass a wide range of emotions. HCPs may experience fear related to various situations after a safety incident. Furthermore, it was also found that some professionals fear future errors, the consequences of errors, lawsuits, litigation or facing patients and their families. Anger may manifest as anger towards oneself or others. Physical distress, anxiety, guilt and a lack of self‐confidence are also common psychological symptoms of safety incidents (Awuah et al. 2024). This review further identifies a range of concepts related to these symptoms. For example, symptoms related to guilt include blame, self‐blame, regret, fault and remorse.

A previous study found that nearly one third of HCPs experienced psychosomatic symptoms such as back pain and headache, along with sleep problems following a safety incident (Mousa et al. 2023). Based on this review, sleep‐related symptoms are the most common symptoms after such an incident. This scoping review also identified symptoms related to the gastrointestinal system, neurological and nervous system, physical pain and tension experienced after a safety incident. Similar results have been reported for tachycardia, increased respiratory rate and muscle tension (Rinaldi et al. 2016).

This study found both positive and negative impacts of patient safety incidents on the lives of HCPs. The negative impacts included reduced professional self‐efficacy, self‐doubt, burnout, intentions to leave the job, absenteeism, mistrust in colleagues, job dissatisfaction, job insecurity and long‐lasting professional grief resulting from involvement in such incidents. The results of this study confirmed the findings of previous research conducted in Finland which showed that HCPs endorsed experiencing reduced professional self‐efficacy, increased turnover intention and absenteeism after making medication errors (Mahat et al. 2024). Similarly, previous studies found that HCPs experienced low confidence in performing procedures, avoiding contact with the affected patient and lost trust in colleagues (Jain et al. 2022). Several studies included in this review also found that social isolation affected the lives of HCPs after experiencing safety incidents. Previous studies have explored the social manifestations of these events, such as avoiding the workplace, isolating oneself, changing one's location and withdrawing from professional settings (Dulko and Zangaro 2022; Zangaro et al. 2022).

Although this scoping review focuses on the symptoms and impacts experienced by HCPs following a safety incident, it is also important to acknowledge that the risk of such events is inherent in healthcare work. According to Strid et al. (2021), professionals are better prepared to handle safety incidents if they have prior experience with risks and potential safety incidents. In contrast, poor preparedness may lead to stress, panic and other negative reactions (Strid et al. 2021). When preparedness fails, it is crucial to recognise the possible impacts of safety incidents on the professionals.

The European Researchers' Network Working on Second Victim (ERNST) has published the Policy Statement on the Second Victim Phenomenon for Increasing Patient Safety. According to ERNST's policy statement, patient safety must be a global priority in health care. Organisations are expected to anticipate and manage risks, and to learn from errors. The statement emphasises the importance of ensuring the functional capacity of HCPs. While the patient must always remain the primary focus, the impact of adverse events on professionals must also be acknowledged. ERNST recommends allocating resources to support SVs, particularly through peer support programmes. Furthermore, a just culture should be fostered within healthcare systems, and the topic should be brought more prominently into public and professional discourse. (Mira et al. 2024) Although this scoping review does not directly promote support for HCPs following a safety incident, it provides valuable insights into the challenges faced by SVs by broadly describing the symptoms and impacts they experience and offering fundamental groundings for organisations to identify them. The findings may also be integrated into the education of healthcare students, enhancing their understanding of the potential implications of safety incidents for professionals.

### 4.1 Implications

This study identified the positive impact of safety incidents on HCPs. These positive impacts require further exploration as they could help to strengthen learning from mistakes and promote patient safety. The findings of this scoping review can be utilised to form a taxonomy, identify the relevant harm to HCPs after a safety incident and increase the understanding of the SV phenomenon and support needs. Based on these findings, an automatic text analysis system could be developed using categorised terms to identify the symptoms and impacts experienced by professionals from adverse event reports. Also, the findings of this scoping review can be utilised in HCPs training and education.

### 4.2 Strengths and Limitations

The strength of this review lies in the rigour of its methodology and further in the large number of identified studies related to the SVS. This extensive body of papers provides the basis for a comprehensive understanding of the symptoms and effects experienced by HCPs after safety incidents. The study was rigorously conducted following the JBI scoping review methodology. The screening process and data extraction were performed by two independent authors, and the data extraction was pilot‐tested by three of the authors. Disagreements were resolved by a research group. These actions strengthen the credibility of the study findings.

Although many studies on SV symptoms and impacts were included, it is possible that some essential studies were inadvertently missing. One potential limitation is that only English‐language studies were included. Many of the included papers were conducted in the USA; only one study was from an African country, which is another limitation of this study.

## 5 Conclusions

This study represents an extensive compilation of the psychological and physical symptoms, and social and professional impacts associated with SVS. It provides a comprehensive and up‐to‐date overview of HCPs' experiences following safety incidents. The categorised terms provided in this study can be utilised in the future in various ways, such as in the training and education of professionals. This review also highlights the often‐overlooked positive impacts of patient safety incidents on HCPs, such as learning from mistakes, increased attentiveness and valuing collegial relationships.

## Author Contributions

Author's involvement in a manuscript were for designing the analysis (L.J., S.M., S.K., T.S., A.W.W., V.J., M.H.), testing the data extraction template (L.J., T.S., M.H.) contributing or selecting data (L.J., S.M., S.K., T.S.), performing the analysis (L.J., S.M., S.K., T.S., M.H.) or writing the manuscript (L.J., S.M.), intellectual comments for the analysis and manuscript (L.J., S.M., S.K., T.S., A.W.W., V.J., M.H.).

## Conflicts of Interest

The authors declare no conflicts of interest.

## Supporting information


**Data S1:** jan70196‐sup‐0001‐Supinfo.docx.

## Data Availability

Data sharing is not applicable to this article as no new data were created or analyzed in this study.
